# Untargeted LC-HRMS Metabolomics and Chemometrics of *Aloe vera* Across Diverse Geographical Origins and Cultivation Practices

**DOI:** 10.3390/plants14111685

**Published:** 2025-05-31

**Authors:** Attilio Anzano, Laura Grauso, Bruna de Falco, Virginia Lanzotti

**Affiliations:** Dipartimento di Agraria, Università di Napoli Federico II, Via Università 100, I-80055 Portici, Italy; attilio.anzano@unina.it (A.A.); laura.grauso@unina.it (L.G.); bruna.defalco@unina.it (B.d.F.)

**Keywords:** *Aloe vera*, chemical composition, natural products, food plant, LC-HRMS, geographical authentication, cocultivation

## Abstract

The chemical composition of *Aloe vera* leaves was investigated by using liquid chromatography–high-resolution tandem mass spectrometry (LC-HRMS/MS). Five *A. vera* samples were collected across diverse geographical origins and cultivation practices: PO (Botanical Garden of Portici, Italy), CAN (Gran Canaria, Canary Islands), CA, E, and MM (Marine Reserve of Torre Guaceto, Brindisi, Italy). Analysis of hydroalcoholic organic extracts revealed 77 organic compounds, including ubiquitous primary metabolites (i.e., sugars, amino acids, and fatty acids) and natural products (i.e., phenols and aromatics, terpenes, and anthraquinones). Principal component analysis (PCA) on the raw dataset indicated a clear separation of samples depending on their geographical origins. PO samples showed high amounts of citric acid, the anthraquinone aloe-emodin, and the alkaloids tropine and tropinone. CAN samples showed high content of malic, citramalic, citraconic, erucic, and 3-dehydroquinic acids. CAN and PO samples contained high amounts of jasmonic, quinic, and 4-methoxycinnamic acids along with aloesin, tyramine, coumarin, and saponarin. Among the Brindisi samples, MM contained high amounts of limonene and α-linolenic acid. CA, E, and MM samples presented high amounts of eritrose-4-phosphate, glucose-1-phosphate, and fructosyl valine along with ginsenoside, proline, and ascorbic acid. These findings indicate that geographical origins and cultivation practices affect to different extents the metabolite profile of *A. vera* plants.

## 1. Introduction

**Figure 1:**
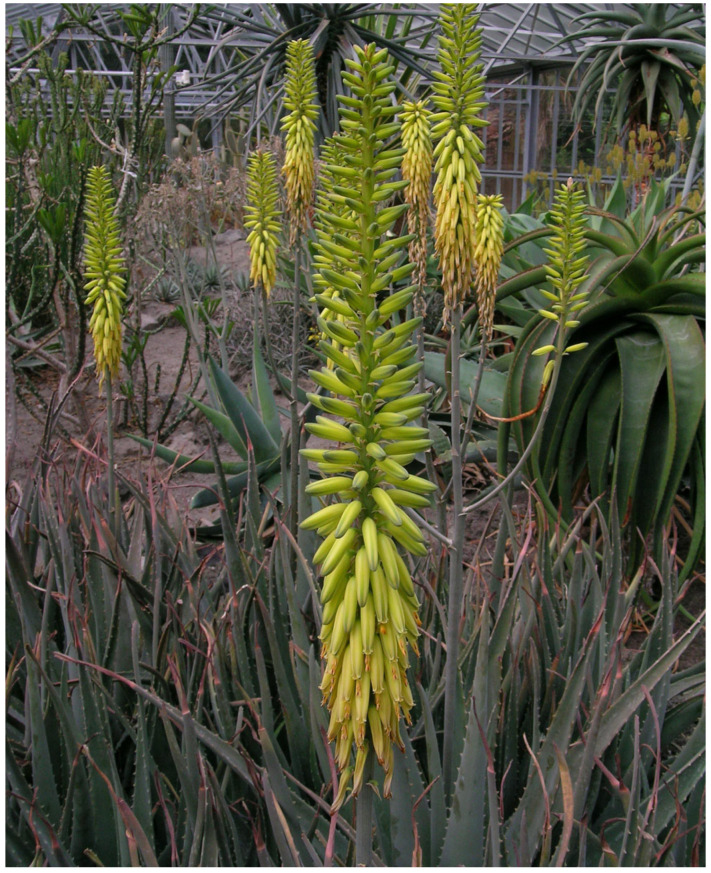
*Aloe vera* plants from the Botanical Garden of Portici, Italy (photo by courtesy of prof. Riccardo Motti).

*Aloe vera* (Figure 1) is a succulent plant well known for its medicinal and cosmetic benefits. It is native to the Arabian Peninsula, and it was introduced to China and various parts of southern Europe, including Italy, Spain, and Portugal, in the 17th century [1]. It is widely naturalized elsewhere, occurring in arid, temperate, and tropical regions on all continents. Its current distribution is the result of human cultivation [2].

This versatile plant is not only a common drug in traditional medicine but a popular ingredient in modern skincare, wellness, and food products [3,4,5,6,7]. In fact, *A. vera* contains several bioactive compounds, including polysaccharides, vitamins, minerals, enzymes, and amino acids, each contributing to its therapeutic effects [1,8,9]. Understanding the chemical profile of *A. vera* can shed light on how its components work synergistically to promote health, treat skin conditions, and support overall well-being.

Recent advances in metabolomics expanded our understanding of the plant’s chemical profile by studying the whole metabolome without long and expensive purification procedures. Recently, this approach has been applied to *A. vera*, identifying several metabolites including phenolics, saponins, terpenes, and anthraquinones [10,11,12]. Among these, aloin and emodin were shown to exhibit antibacterial, anticancer, antiviral, anti-inflammatory, and analgesic activities [13,14,15].

In this article, we explored the chemical profile of samples of *A. vera* from diverse geographical origins and cultivation practices by using untargeted LC-HRMS. This method allowed the analysis of the whole plant metabolome through the detection of major and minor compounds in the plant extracts. The use of the mass tool program Compound Discoverer aided compound annotation by comparing the obtained data with those of standard mass libraries [16]. Further, chemometrics, including PCA, OPLS-DA, and hierarchical clustering analyses, were used to compare the different plant samples to evaluate differences in their metabolite profiles.

## 2. Results

### Metabolomics Analysis and Compound Annotation

**Figure 2:**
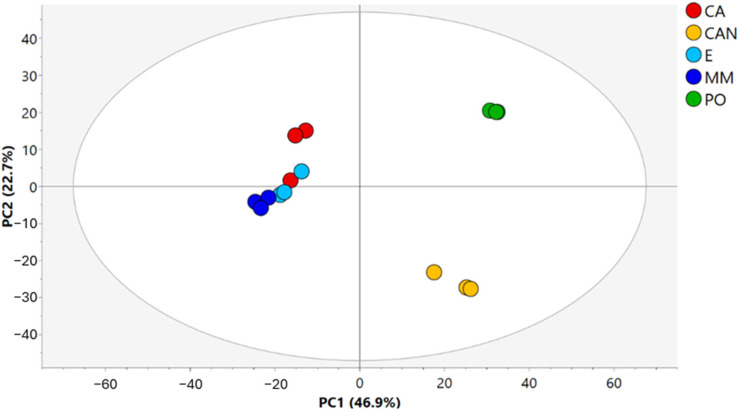
PCA score plot of *A. vera* samples.

Our study focused on *A. vera* leaves from diverse geographical origins: PO (collected in the Botanical Garden of Portici, Italy), CAN (collected in Gran Canaria, Canary Islands), CA, E, and MM (collected in the Marine Reserve of Torre Guaceto, Brindisi, Italy). We performed a comprehensive analysis of the hydroalcoholic extracts of leaves using liquid chromatography–high-resolution tandem mass spectroscopy (LC-HRMS/MS). This MS-based metabolomic analysis allowed us to explore the metabolite profiles in detail, uncovering potential changes among the plants collected in different geographical origins and cultivations practices, and evidencing eventually unique features of each plant extract. The analysis was performed in positive ionization mode, and the raw dataset was analyzed using unsupervised principal component analysis (PCA) (Figure 2).

The resulting PCA score plot of *A. vera* samples, shown in Figure 2, accounted for 69.6% of the total variance, with principal component 1 explaining 46.9% and principal component 2 explaining 22.7%. The data indicated a clear separation of samples depending on the geographical origins. In fact, the first component clearly separated CAN (Grand Canaria, Spain) and PO (Portici, Italy) samples from CA, E, and MM (Brindisi, Italy) samples. The second component discriminated between CAN and PO samples. A minor difference in the second component appeared in two replicates of CA compared with the other samples from Brindisi.

The Brindisi samples differed in cultivation practices: CA samples were cultivated alone in open field conditions, E samples were cocultivated with *Eucalyptus* plants, and MM samples were cocultivated with mixed plants of Mediterranean area. Thus, the obtained data indicated a low separation of samples based on agricultural practices (Figure 2).

The data obtained by LC-MS were analyzed to obtain metabolite annotation to find the compounds responsible for the observed sample separations. This was performed using the Compound Discoverer 3.3 software (Thermo Fisher Scientific, Waltham, MA, USA). Figure S1 shows a detailed description of the workflow used. Metabolite annotation was obtained by matching the accurate masses and the fragmentation patterns of the detected peaks in the LC-MS chromatograms (Figures S2 and S3) with data available from analytical standards and online libraries, including the mzCloud fragmentation database.

Confidence in metabolite identification was assigned from level 1 to 4 following the Metabolomics Standards Initiative (MSI) guidelines. Thus, metabolites identified using RT, *m*/*z*, and/or MS/MS from reference standards were assigned to level 1. Metabolites annotated using *m*/*z* and MS/MS from spectral libraries without reference standards were assigned to level 2, putatively characterized metabolite classes using *m*/*z* were level 3, and unknown metabolites, if any, were classified as level 4 [17].

A total of 77 organic compounds were annotated: 32 metabolites were identified using exact masses and RT of authentic standards (level 1); 24 metabolites were putatively matched based on MS/MS fragmentation patterns (level 2); and 21 metabolites were included in a class of compounds (level 3) (Table 1). The identified compounds included ubiquitous primary metabolites, i.e., sugars, amino acids, and fatty acids, and natural compounds, i.e., phenols and aromatics, terpenes, and anthraquinones, the latter being typically present in aloe species.

**Figure 3:**
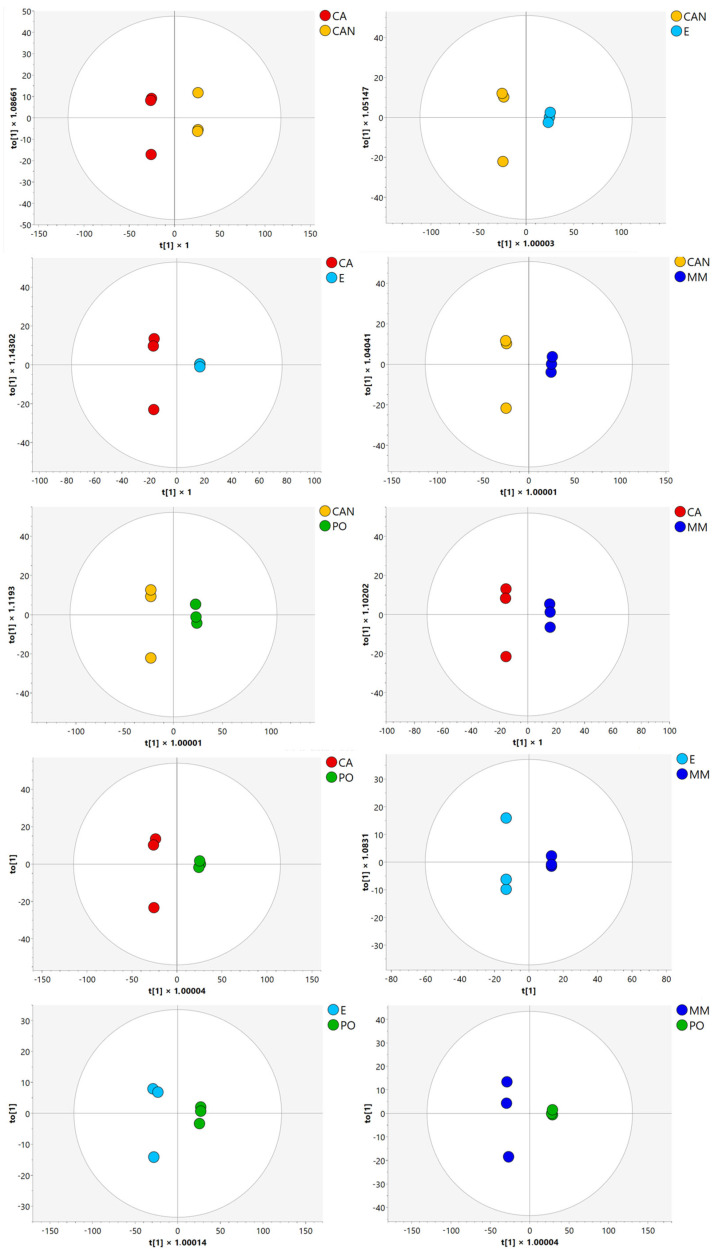
OPLS-DA of *A. vera* samples.

Then, orthogonal partial least squares discriminant analysis (OPLS-DA) (Figure 3) and variable importance in projection analysis (VIP) helped to further identify the metabolites with the highest discriminative power, highlighting the molecules that contributed most significantly to the observed differences among the samples.

Quantitative data on the identified metabolites were obtained by peak integration. Table S1 lists the average areas and the standard deviations for the detected metabolites.

**Figure 4:**
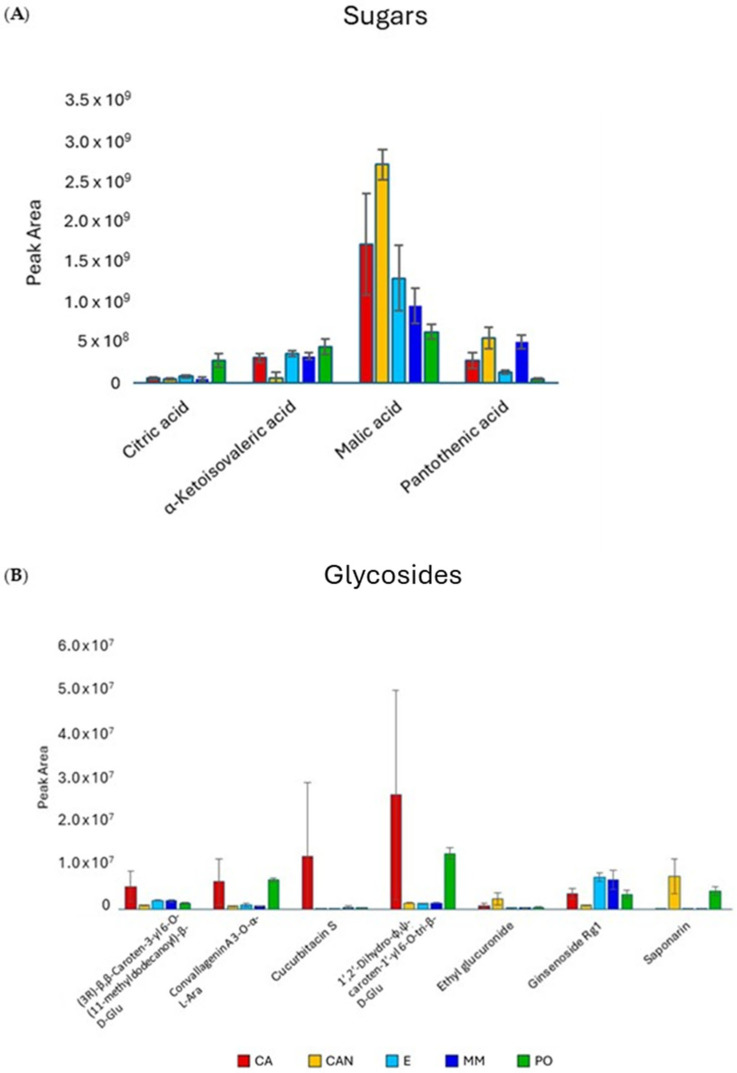
Quantitative analysis of sugars (**A**) and glycosides (**B**) identified in *A. vera* extracts. CA, E, MM (Torre Guaceto); CAN (Gran Canaria); PO (Portici). Displayed data refer to the mean and standard deviation of three replicates.

The most abundant compounds in the extracts were sugars, on which the quantitative data are reported in Figure 4A. Fructosyl valine was the predominant sugar in CA and MM samples, while glucose-1-phosphate and eritrose-4-phosphate were present at higher amounts in CA, E, and MM samples.

The analysis of glycosides (Figure 4B) did not show significant variation among samples except for CAN and PO, which were found to be richer in saponarin than CA, E, and MM.

**Figure 5:**
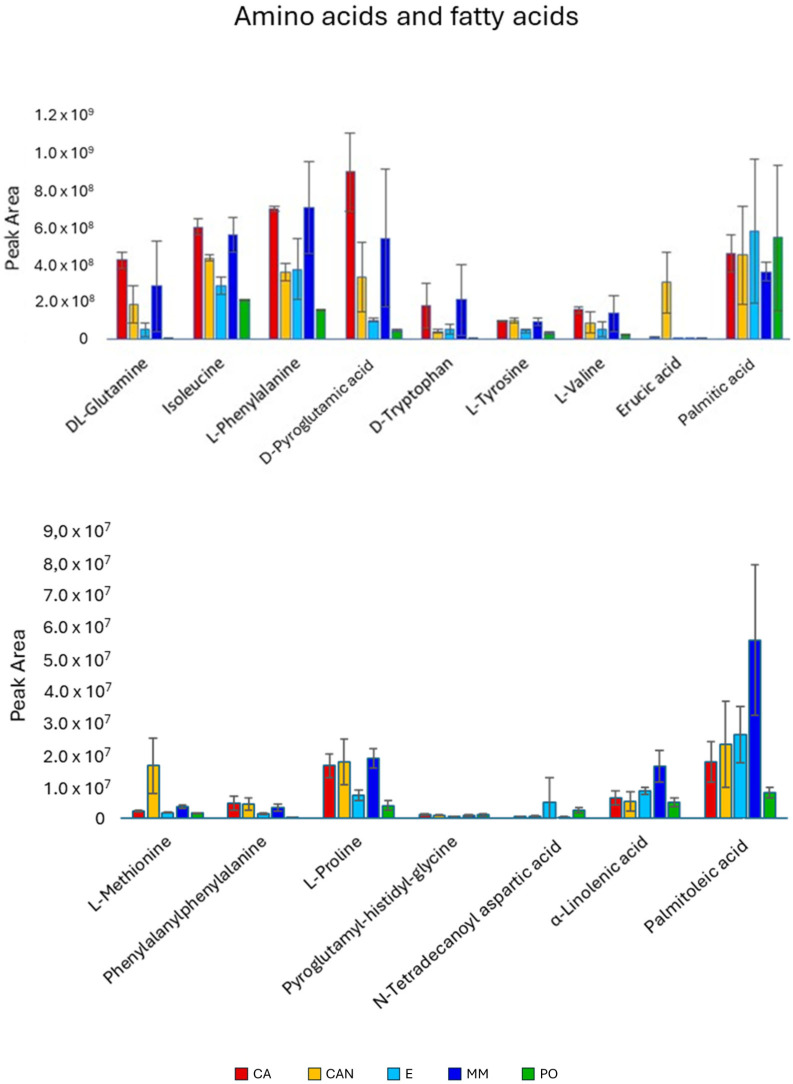
Quantitative analysis of amino acids and fatty acids identified in *A. vera* extracts. CA, E, MM (Torre Guaceto); CAN (Gran Canaria); PO (Portici). Displayed data refer to the mean and standard deviation of three replicates.

Amino acids (Figure 5) were generally found at higher amounts in CA and MM. The quantities of glutamine, isoleucine, and phenylalanine were significantly higher in CA than in CAN, E, and PO. The same trend was also observed for pyroglutamic acid and tryptophan. A high accumulation of methionine was noticed in the CAN sample, significantly more than in all the other samples.

The fatty acids (Figure 5) did not considerably impact the separation of the sample set. Palmitic acid did not show significant differences among the different samples. However, erucic acid was significantly higher in CAN than in the other samples, while palmitoleic and linolenic acids were predominant in MM, with palmitoleic acid being relatively lower in PO.

**Figure 6:**
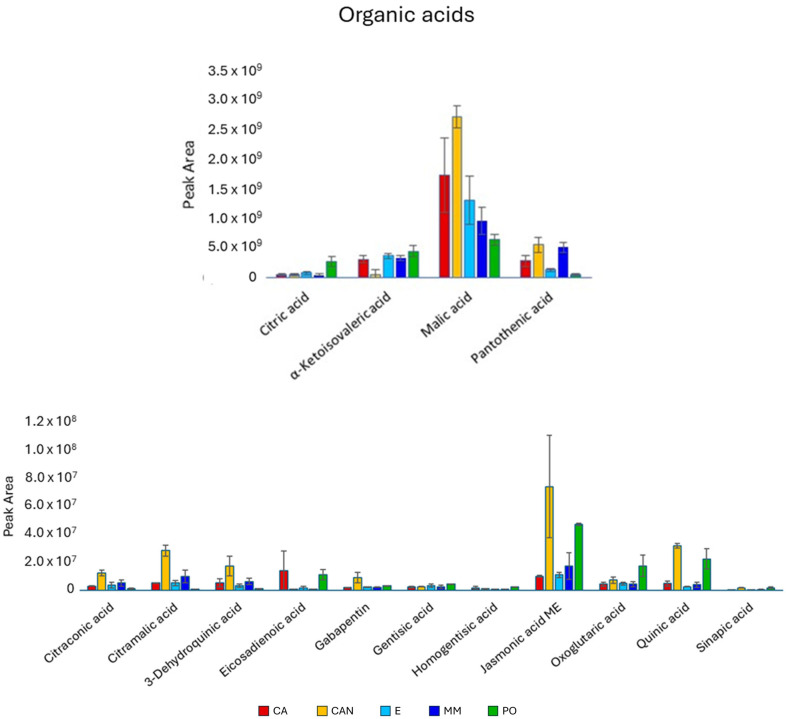
Quantitative analysis of organic acids identified in *A. vera* extracts. CA, E, MM (Torre Guaceto); CAN (Gran Canaria); PO (Portici). Displayed data refer to the mean and standard deviation of three replicates.

Regarding organic acids (Figure 6), CAN presented the highest content of them. Malic acid was the most abundant metabolite found in our analysis, and it was significantly higher in CAN than in the other samples. The same trend was found for citramalic acid, predominating in CAN. Quinic acid was significantly higher in CAN and PO than in MM, CA and E, while jasmonic acid methyl ester was significantly higher in CAN than in CA and E, but not when compared with MM and PO.

**Figure 7:**
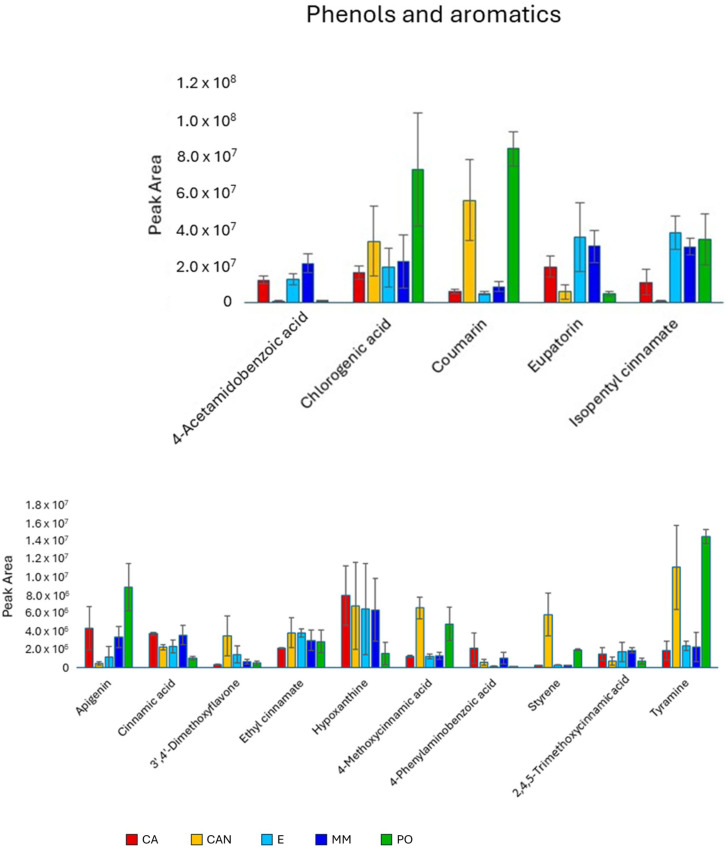
Quantitative analysis of phenols and aromatics identified in *A. vera* extracts. CA, E, MM (Torre Guaceto); CAN (Gran Canaria); PO (Portici). Displayed data refer to the mean and standard deviation of three replicates.

Among phenols and aromatic compounds (Figure 7), we observed significantly higher content of chlorogenic acid in PO than in E and MM and of coumarin in PO and CAN than in MM, CA, and E. Acetoamidobenzoic acid and eupatorin were the only two aromatic compounds less abundant in CAN and PO than in MM, CA, and E. Significantly larger quantities of styrene, tyramine, and methoxycinnamic acid were found in CAN and PO than in MM, CA, and E.

**Figure 8:**
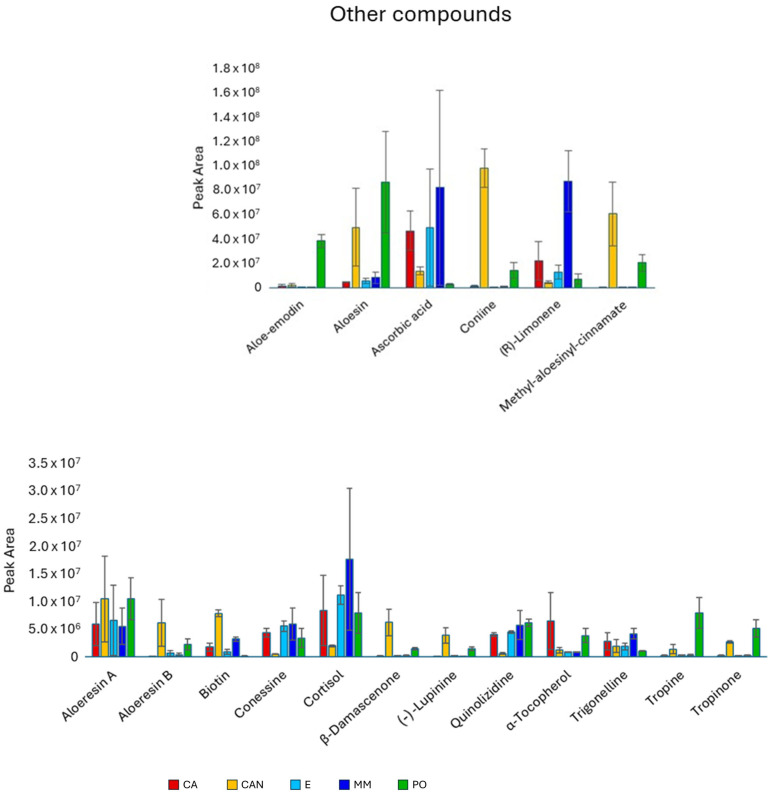
Quantitative analysis of other compounds, including alkaloids, anthraquinones, terpenes, and vitamins, identified in *A. vera* extracts. CA, E, MM (Torre Guaceto); CAN (Gran Canaria); PO (Portici). Displayed data refer to the mean and standard deviation of three replicates.

Concerning other compounds (Figure 8), the antraquinone aloe-emodin predominated in PO compared with the other samples, while the C-glycosilated chromone aloesin was more abundant in PO than in CA, E, and MM. Coniine and methyl aloesinyl cinnamate were both significantly more abundant in CAN and PO, since they were nearly absent in CA, E, and MM. The monoterpene limonene showed high abundancy in MM. The quantity of the vitamin B8 (biotin) was significantly higher in CAN.

The alkaloid lupinine showed significantly higher content in CAN and PO than in CA, E, and MM. The two alkaloids tropine and tropinone significantly predominated in PO. Finally, tropinone was more abundant in CAN than in CA, E, and MM.

**Figure 9:**
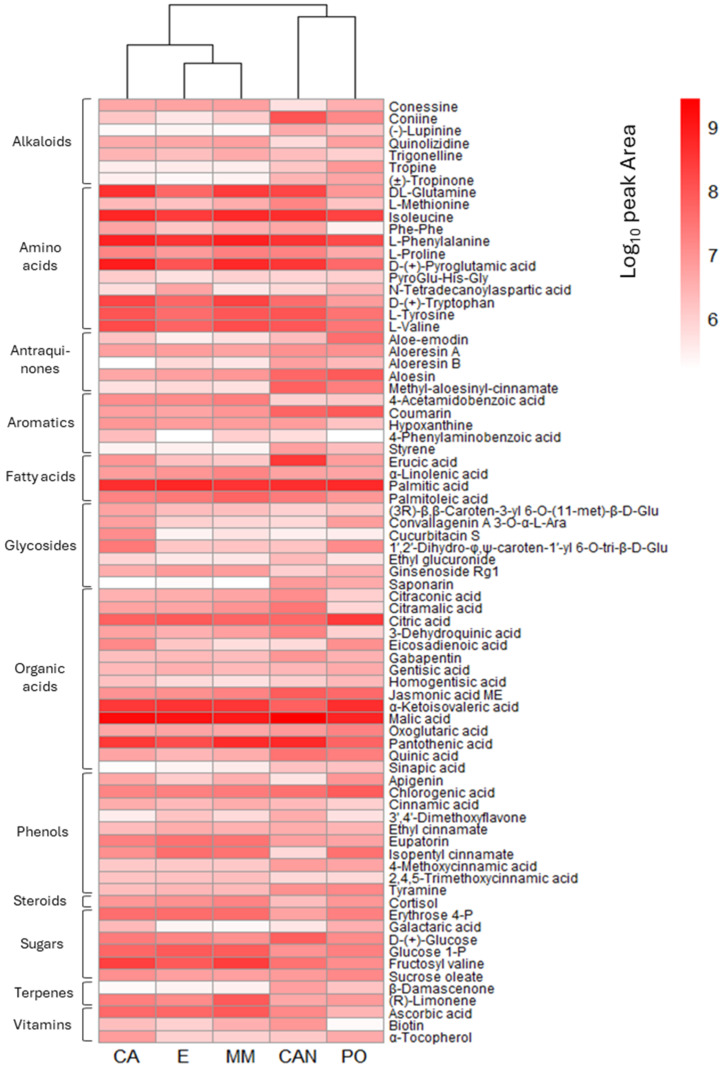
Heatmap representing the average peak area of the detected metabolites at each growing condition (log_10_ scaled, replicates = 3). Hierarchical cluster analysis was performed, and the dendrogram is shown on the upper part of the heatmap.

The obtained quantitative data are shown in the heatmap reported in Figure 9, where the relative quantities of the 77 detected metabolites are displayed to easily compare the abundance of the metabolites in the different samples. To obtain a better visualization, the data used in the heatmap were log_10_ scaled. The hierarchical cluster analysis performed on the selected metabolites confirmed the sample discrimination observed in the PCA, highlighting the presence of the two separated groups of samples.

## 3. Discussion

Despite *A. vera* being a widely diffused commercial plant, there are only a few articles focusing on metabolomic analysis of the gel obtained from the plant leaves. Most works employed nuclear magnetic resonance (NMR) spectroscopy, and only a few performed untargeted analyses based on LC-MS/MS techniques. Two studies focusing on NMR spectroscopy reported relatively simple compounds, including amino acids and phenylpropanoids such as chlorogenic acid and benzoic acid [11,18]. However, LC-MS approaches were able to identify a wider variety of metabolites. An article published by Breaud et al. (2023) identified several compounds found by our study as well, such as chlorogenic acid, aloesin, linolenic acid, and apigenin derivatives [19]. Lee et al. (2013) employed UPLC-ESI-MS to identify several metabolites, reporting the anthraquinone aloe-emodin to vary among the treatments they applied to the plant [20]. In a previous work, Lee et al. (2012) used UPLC-Q-TOF-MS and identified several metabolites that were also found in this study, such as aloesin, malic acid, pyroglutamic acid, glucose, glucuronic acid, and sucrose (here found as sucrose oleate) [10]. In our work, 77 different metabolites were annotated and then used to highlight the differences between aloe plants from different geographical origins and cultivation practices.

Geographical characterization of plants grown in different areas through metabolomics has been used in the past, and the differences in metabolite profiles have been described. For instance, in the work of Lee et al. (2015), 284 samples of tea collected from different areas of China, Japan, and South Korea were chemically characterized through NMR spectroscopy, and the resulting PCA was able to correctly reproduce the three main geographical locations [21]. Another research group characterized the lipidome of tobacco leaves coming from three different regions of China and found correlations between the lipidome composition, the geographical position, and other environmental factors such as the growth temperature of the tobacco plant in that area [22].

Our work is in line with these findings. In fact, the data we obtained were able to clearly distinguish between plants that were grown in different geographical locations (Brindisi, Grand Canaria, and Portici). Fewer differences were found in relation to agricultural practices based on mixed cultivation within the same geographical area. Scientific literature about the impact of cocultivation of different plants on their metabolome is still lacking, as there are no studies that review the interactions between two or more plants from a metabolomic point of view. A study from Pedersen et al. (2013) found that 34 metabolites increased and 54 decreased, the latter including aromatic and branched amino acids, upon cocultivation of *Arabidopsis thaliana* with *Trifolium repens,* suggesting that *T. repens* was in some way responsible for a shift in the secondary metabolite content of *A. thaliana* [23]. However, our results did not highlight major differences among the primary and secondary metabolites of *A. vera* cocultivated with other plant species.

It is well known that origin affects the metabolic profile of plants. This aspect must be considered because it could influence the quantity of specific metabolites and therefore the biological activity of the extract. Knowing the composition of plant extracts of different sources could be a good starting point to obtain enriched natural extracts with higher biological activity. Thus, our study has shown the potential of applying an untargeted metabolomics approach to explore metabolomic differences in *A. vera* samples according to geographical regions of production.

## 4. Materials and Methods

### 4.1. Chemicals

First-grade methanol and formic acid were purchased from Delchimica Scientific Laboratories Glassware (Naples, Italy). Pure standard amino acids, organic acids, phenolics, and nucleosides were used as references (Sigma-Aldrich, Milano, Italy).

### 4.2. Plant Material

*Aloe vera* samples were collected and analyzed in triplicates. The samples were harvested in different geographical areas and in different cultivation conditions.

**CA**: harvested in open field and collected at the organic farm MATER, Torre Guaceto Marine Reserve, Brindisi, Italy.

**CAN**: harvested in open field in Gáldar, Gran Canaria, Spain.

**E**: harvested in open field with mixed vegetation of *Eucalyptus* plants and collected at the organic farm MATER, Torre Guaceto Marine Reserve, Brindisi, Italy.

**MM**: harvested in open field with mixed vegetation of Mediterranean plants and collected in at the organic farm MATER, Torre Guaceto Marine Reserve, Brindisi, Italy.

**PO**: harvested in the Botanical Garden, Real Palace of Portici, Portici, Italy.

Three leaves were harvested in the field from 3 adult plants for each geographical location. The external green layer was removed using a kitchen knife, and the internal pulp (aloe gel) was collected in a tube and frozen until further extraction.

### 4.3. Metabolite Extraction Procedure

The frozen gels were lyophilized (Zirbus laboratory freeze-drier) for 2 days to remove the water content, and therefore, around 200 mg of gel was extracted using 50 mL of a methanol/water solution (70:30), continuously stirring for 1 h. Then, the extracts were centrifuged (4000 rpm for 10 min) and filtrated through filter paper, and the solutions were dried by using a rotary evaporator. An aliquot (10 mg) of each dried sample was collected in 4 mL glass vial, and the content was dried using a rotational vacuum concentrator (RVC 2-18 CDplus, CHRIST, Osterode am Harz, Germany). The vials were stored at 4 °C until the LC-MS analysis was performed. Three biological replicates were prepared for each sample group. Validation of the extraction protocol was obtained by using a standardized sample preparation protocol previously developed and applied for plant analysis [24,25,26,27]. This allowed building compound libraries that enabled effective compound identification and efficient dereplication. This protocol was based on the use of solvents (methanol and water) complementary for polarity, allowing a broad range of organic compounds to be identified. The use of blank samples and quality standard samples allowed the validation of the applied method by ensuring the reliability and accuracy of measurements.

### 4.4. LC-HRMS and LC-HRMS/MS Analyses

Liquid chromatography–high-resolution mass spectrometry (LC-HRMS) and high-resolution tandem mass spectrometry (HR-MS/MS) were performed using a Thermo LTQ Orbitrap XL mass spectrometer (Thermo Fisher Scientific Spa, Rodano, Italy) coupled to a Thermo U3000 HPLC system. 5 µL of sample were injected in a Kinetex C18 column (5 µm, 50 × 2.1 mm, Phenomenex, Torrance, CA, USA), with a flowrate of 0.2 mL/min, to separate the individual metabolites. The gradient elution, 0.1% formic acid in H_2_O (solvent A) and CH_3_OH (solvent B), was optimized as follows: 5% B at 1 min, 5–100% B over 40 min, held at 100% B for 10 min. HR-MS and HR-MS/MS spectra were acquired in positive ion mode selecting a *m*/*z* range of 100–2000 with 60,000 resolution to generate data-dependent scans for identification. The MS parameters were as follows: spray voltage 4.80 kV, capillary temperature 285 °C, sheath gas rate 32.0 units N_2_ (ca. 150 mL/min), auxiliary gas rate 15 units N_2_ (ca. 50 mL/min). To prevent the formation of cluster ions while causing no actual fragmentation, source fragmentation was enabled using a mild potential of 35 V to avoid formation of clusters and avoidance of ion fragmentation. The MS/MS spectra of the selected ions were collected with collision-induced dissociation (CID) fragmentation, wideband activation mode, using the following parameters: isolation width ±3.00 Da, collision energy 35 units, activation Q 0.250 units, and activation time 30 ms.

### 4.5. Data Analysis and Metabolite Annotation

The data obtained from LC-MS were analyzed through the software Compound Discoverer 3.3 (Thermo Fisher Scientific, Hemel Hempstead, UK). The annotation of the metabolites was performed by matching accurate masses and fragmentation patterns to analytical standards and online libraries, including the mzCloud fragmentation database. The confidence in metabolite identification was assigned into levels 1–4 following the Metabolomics Standards Initiative (MSI) guidelines [28,29]. The retention time (RT) range applied was 0–40 min, the mass range was *m*/*z* 70–1500, the mass tolerance for peak picking and metabolite annotation was set to less than 5 ppm for both precursor and fragment ions, and the maximum retention time shift was 0.25 min. The multivariate data analysis was conducted using SIMCA18 (Sartorius, Gottingen, Germany). The datasets were log-transformed and Pareto scaled before model creation. The data were first explored using unsupervised principal component analysis (PCA). Subsequently, orthogonal partial least squares discriminant analysis (OPLS-DA) was performed to highlight the metabolites causing the differences among the treatments. Scatter plots, loading plots, and variable importance in projection (VIP) score plots were generated and interpreted to identify significantly altered metabolites between the control and treatment groups. Metabolites with VIP scores greater than 1 were considered strong contributors. The quality of models was validated through cross-validation using the leave-one-out method, assessing the goodness of fit of the model (R2X) for PCA, (R2Y) for OPLS-DA, and predictive ability (Q2) values. A *p*-value less than 0.05 was considered to define statistical significance.

## 5. Conclusions

In conclusion, the untargeted LC-HRMS/MS metabolomics of *A. vera* from diverse geographical origins and cultivation practices highlight the remarkable diversity and complexity of its biochemical composition. Our study demonstrated that variations in metabolite profiles were greatly influenced by environmental factors, while less influence was found by cultivation practices. Therefore, our analytical approach could constitute a powerful model to analyze geographical distribution of plants. The differences observed in *A. vera* samples from different geographical areas could have implications for the plant’s medicinal and therapeutic properties, suggesting that region-specific formulations may offer distinct health benefits. Further research integrating metabolomics of *A. vera* with bioactivity studies can lead to a deeper understanding of its potential and pave the way for more targeted and effective use in both traditional and modern medicine. By integrating advanced technologies and cross-country studies, we can unlock the full potential of this versatile plant, ensuring its sustainable use and optimal exploitation for health and wellness applications worldwide.

## Figures and Tables

**Table 1:** Metabolites identified in *A. vera* samples through LC-HRMS analysis in positive ion mode.

N.	Compound Class	Compound Annotation	Formula	RT (min)	[M+H]^+^	ppm	MS/MS	Id. Level
**1**	Alkaloids	Conessine	C_24_H_40_N_2_	26.75	357.3263	−0.24	n.a.	3
**2**	Alkaloids	Coniine	C_8_H_17_N	14.90	128.1434	0.02	82.09/69.07/55.05	2
**3**	Alkaloids	(−)-Lupinine	C_10_H_19_NO	16.51	170.1539	−0.23	157.02/128.14	2
**4**	Alkaloids	Quinolizidine	C_9_H_17_N	4.00	140.1434	0.19	102.55/58.06	2
**5**	Alkaloids	Trigonelline	C_7_H_7_NO_2_	4.10	138.0550	0.10	n.a.	3
**6**	Alkaloids	Tropine	C_8_H_15_NO	2.41	142.1227	0.42	124.11/67.05/43.02	2
**7**	Alkaloids	(±)-Tropinone	C_8_H_13_NO	6.39	140.1070	−0.01	98.09/81.07	2
**8**	Amino acids	DL-Glutamine	C_5_H_10_N_2_O_3_	1.50	147.0765	0.20	130.05/84.04	1
**9**	Amino acids	L-Methionine	C_5_H_11_NO_2_S	1.62	150.0584	0.13	n.a.	1
**10**	Amino acids	Isoleucine	C_6_H_13_NO_2_	1.97	132.1019	0.23	86.10/69.07	1
**11**	Amino acids	Phenylalanylphenylalanine	C_18_H_20_N_2_O_3_	15.61	313.1546	−0.14	120.08/103.05	1
**12**	Amino acids	L-Phenylalanine	C_9_H_11_NO_2_	3.01	166.0862	−0.25	120.08/103.05	1
**13**	Amino acids	L-Proline	C_5_H_9_NO_2_	2.32	116.0706	−0.08	99.05/84.04/56.05	1
**14**	Amino acids	D-(+)-Pyroglutamic acid	C_5_H_7_NO_3_	1.78	130.0499	0.29	130.05/84.04	1
**15**	Amino acids	Pyroglutamyl-histidyl-glycine	C_13_H_17_N_5_O_5_	1.92	324.1289	−4.19	n.a.	3
**16**	Amino acids	N-Tetradecanoylaspartic acid	C_18_H_33_NO_5_	21.23	344.2431	−0.28	n.a.	3
**17**	Amino acids	D-(+)-Tryptophan	C_11_H_12_N_2_O_2_	6.21	205.0971	−0.21	188.07/146.06/91.05	1
**18**	Amino acids	L-Tyrosine	C_9_H_11_NO_3_	1.99	182.0811	−0.18	165.05/136.08/123.04	1
**19**	Amino acids	L-Valine	C_5_H_11_NO_2_	1.56	118.0862	−0.12	72.08/55.05	1
**20**	Antraquinones	Aloe-emodin	C_15_H_10_O_5_	25.81	271.0601	−0.03	253.05/241.05/225.06	1
**21**	Antraquinones	Aloeresin A	C_28_H_28_O_11_	17.77	541.1702	−0.53	275.09/147.04	1
**22**	Antraquinones	Aloeresin B	C_19_H_22_O_9_	12.91	395.1335	−0.81	233.08/191.07	1
**23**	Antraquinones	Aloesin	C_19_H_22_O_9_	16.04	395.1334	−0.48	233.08/203.08/115.04	1
**24**	Antraquinones	Methyl aloesinyl cinnamate	C_29_H_32_O_10_	22.09	541.2067	−0.31	497.18/217.06/131.05	1
**25**	Aromatics	4-Acetamidobenzoic acid	C_9_H_9_NO_3_	3.58	180.0630	−13.85		1
**26**	Aromatics	Coumarin	C_9_H_6_O_2_	15.51	147.0441	0.13	119.05/91.05/65.04	1
**27**	Aromatics	Hypoxanthine	C_5_H_4_N_4_O	2.41	137.0458	0.12	119.03/55.03	1
**28**	Aromatics	4-Phenylaminobenzoic acid	C_13_H_11_NO_2_	10.04	214.0862	−0.08	203.09/180.06	1
**29**	Aromatics	Styrene	C_8_H_8_	3.26	105.0698	−0.56	95.05/79.05	1
**30**	Fatty acids	Erucic acid	C_22_H_42_O_2_	35.77	339.3257	−0.30	321.31/303.30	1
**31**	Fatty acids	α-Linolenic acid	C_18_H_30_O_2_	34.23	301.2137	−0.24		2
**32**	Fatty acids	Palmitic acid	C_16_H_32_O_2_	26.12	274.2740 ^a^	−0.20	256.26/88.07	1
**33**	Fatty acids	Palmitoleic acid	C_16_H_30_O_2_	30.67	277.2162 ^b^	−0.20	259.20/235.17	1
**34**	Glycosides	(3R)-β,β-Caroten-3-yl 6-O-(11-methyldodecanoyl)-β-D-glucopyranoside	C_59_H_90_O_7_	39.60	911.6782	2.43	n.a.	3
**35**	Glycosides	Convallagenin A 3-O-α-L-arabinopyranoside	C_32_H_52_O_9_	35.64	581.3657	−4.74	n.a.	3
**36**	Glycosides	Cucurbitacin S	C_30_H_42_O_6_	25.69	499.3053	−0.3	481.29/283.20/203.05	2
**37**	Glycosides	1′,2′-Dihydro-φ,ψ-caroten-1′-yl 6-O-tridecanoyl-β-D-glucopyranoside	C_59_H_88_O_7_	38.856	909.6635	3.55	n.a.	3
**38**	Glycosides	Ethyl glucuronide	C_8_H_14_O_7_	2.38	223.0812	−0.34	203.08/177.48	2
**39**	Glycosides	Ginsenoside Rg1	C_42_H_72_O_14_	36.27	801.4961	−4.23	n.a.	3
**40**	Glycosides	Saponarin	C_27_H_30_O_15_	16.38	595.1657	0.58	n.a.	3
**41**	Organic acids	Citraconic acid	C_5_H_6_O_4_	2.05	131.0339	0.20	86.10/71.01	2
**42**	Organic acids	Citramalic acid	C_5_H_8_O_5_	2.11	149.0445	−0.09	113.02/103.04/71.01	2
**43**	Organic acids	Citric acid	C_6_H_8_O_7_	1.82	215.0162	−0.01	203.08/170.81/72.08	1
**44**	Organic acids	3-Dehydroquinic acid	C_7_H_10_O_6_	5.82	191.0549	−0.18	105.01/87.00/69.03	2
**45**	Organic acids	Eicosadienoic acid	C_20_H_36_O_2_	35.62	309.2788	0.09	n.a.	3
**46**	Organic acids	Gabapentin	C_9_H_17_NO_2_	12.06	194.1151	−0.37	150.01/144.99/113.10	2
**47**	Organic acids	Gentisic acid	C_7_H_6_O_4_	2.98	155.0339	−0.14	n.a.	3
**48**	Organic acids	Homogentisic acid	C_8_H_8_O_4_	11.61	169.0495	−0.44	n.a.	3
**49**	Organic acids	Jasmonic acid methyl ester	C_13_H_20_O_3_	11.86	225.1485	−0.29	203.08/149.10/99.04	2
**50**	Organic acids	α-Ketoisovaleric acid	C_5_H_8_O_3_	1.79	117.0546	−0.07	84.96/55.05	2
**51**	Organic acids	Malic acid	C_4_H_6_O_5_	1.58	157.0108	0.08	121.65/93.90	1
**52**	Organic acids	Oxoglutaric acid	C_5_H_6_O_5_	1.85	147.0289	0.33	n.a.	3
**53**	Organic acids	Pantothenic acid	C_9_H_17_NO_5_	3.88	220.1179	−0.44	184.09/90.05	1
**54**	Organic acids	Quinic acid	C_7_H_12_O_6_	1.65	193.0706	−0.16	n.a.	3
**55**	Organic acids	Sinapic acid	C_11_H_12_O_5_	13.32	225.0758	0.11	n.a.	3
**56**	Phenols	Apigenin	C_15_H_10_O_5_	23.46	271.0601	−0.11	n.a.	3
**57**	Phenols	Chlorogenic acid	C_16_H_18_O_9_	12.18	355.1022	−0.26	259.05/235.06/205.04	1
**58**	Phenols	Cinnamic acid	C_9_H_8_O_2_	3.01	149.0597	−0.04	n.a.	3
**59**	Phenols	3′,4′-Dimethoxyflavone	C_17_H_14_O_4_	19.89	283.0964	−0.16	251.08/241.08/203.08	1
**60**	Phenols	Ethyl cinnamate	C_11_H_12_O_2_	11.36	177.0910	−0.42	157.25/149.02/109.73	2
**61**	Phenols	Eupatorin	C_18_H_16_O_7_	16.39	345.0968	−0.16	327.08/285.07/267.06	2
**62**	Phenols	Isopentyl cinnamate	C_14_H_18_O_2_	24.64	219.1379	−0.07	230.08/177.09/164.08	1
**63**	Phenols	4-Methoxycinnamic acid	C_10_H_10_O_3_	14.03	179.0702	−0.48	123.04/105.03/95.04	1
**64**	Phenols	2,4,5-Trimethoxycinnamic acid	C_12_H_14_O_5_	10.29	239.0916	0.91	n.a.	3
**65**	Phenols	Tyramine	C_8_H_11_NO	2.54	138.0914	0.16	96.07/94.06/80.04	2
**66**	Steroids	Cortisol	C_21_H_30_O_5_	30.94	363.2142	−6.74	331.22/203.08	2
**67**	Sugars	Erythrose 4-P	C_4_H_9_O_7_P	1.39	201.0160	0.54	158.99/98.97/56.96	2
**68**	Sugars	Galactaric acid	C_6_H_10_O_8_	5.65	211.0444	−2.12	n.a.	3
**69**	Sugars	D-(+)-Glucose	C_6_H_12_O_6_	1.43	198.0972 ^a^	0.10	145.04/127.02/85.02	1
**70**	Sugars	Glucose 1-P	C_6_H_13_O_9_P	1.59	261.0370	0.09	244.11/216.12/203.08/	2
**71**	Sugars	Fructosyl valine	C_11_H_21_NO_7_	1.61	280.1390	−0.25	262.12/244.11/216.12	2
**72**	Sugars	Sucrose oleate	C_30_H_54_O_12_	1.65	304.1867	−4.55	n.a.	3
**73**	Terpenes	β-Damascenone	C_13_H_18_O	11.71	191.1430	−0.64	173.17/121.10/107.08	2
**74**	Terpenes	(R)-Limonene	C_10_H_16_O_3_	22.30	207.0992	−0.09	149.09/97.02	2
**75**	Vitamins	Ascorbic acid	C_6_H_8_O_6_	2.68	199.0213	0.04	149.02/87.00/56.01	1
**76**	Vitamins	Biotin	C_10_H_16_N_2_O_3_S	12.88	245.0954	−0.33	227.05/203.85	2
**77**	Vitamins	α-Tocopherol	C_29_H_50_O_2_	37.45	431.3883	−0.32	n.a.	3

^a^ [M + H_2_O]^+^; ^b^ [M + Na]^+^

## Data Availability

The raw data supporting the conclusions of this article will be made available by the authors on request.

## Supplementary Materials

The following supporting information can be downloaded at: https://www.mdpi.com/article/10.3390/plants14111685/s1, Table S1. Relative area of the compounds identified through LC-MS analysis. Data are shown as an average of three replicates and the relative standard deviation. Figure S1. Description of the workflow used in Compound Discoverer 3.3, including key nodes and parameters used to filter compounds. Figure S2. Representative LCs of *A. vera* extracts: PO (Portici, Italy); CA (Torre Guaceto, Italy); CAN (Gran Canaria, Spain); E (Torre Guaceto, Italy); MM (Torre Guaceto, Italy). Figure S3. EICs of identified compounds of *A. vera* extracts with their MS/MS fragmentations.
