# Supplementary material for: Untargeted LC-HRMS Metabolomics and Chemometrics of Aloe vera Across Diverse Geographical Origins and Cultivation Practices
Source: Plants (Basel). 2025 May 31;14(11):1685. doi: 10.3390/plants14111685 (PMC12157740; doi:10.3390/plants14111685)

Untargeted LC-HRMS Metabolomics and Chemometrics of *Aloe vera* Across Diverse Geographical Origins and Cultivation Practices

Attilio Anzano, Laura Grauso, Bruna de Falco and Virginia Lanzotti *

Dipartimento di Agraria, Università di Napoli Federico II, Via Università 100, I-80055 Portici, Naples, Italy; attilio.anzano@unina.it (A.A.); laura.grauso@unina.it (L.G.); bruna.defalco@unina.it (B.d.F.)

- Correspondence: virginia.lanzotti@unina.it (V.L.), tel.: +390812539459

SUPPLEMENTARY MATERIAL

Table S1. Relative area of the compounds identified through LC-MS analysis. Data are shown as an average of three replicates and the relative standard deviation.

Figure S1. Description of the workflow used in Compound Discoverer 3.3, including key nodes and parameters used to filter compounds.

Figure S2. Representative LCs of *A. vera* extracts: PO (Portici, Italy); CA (Torre Guaceto, Italy); CAN (Gran Canaria, Spain); E (Torre Guaceto, Italy); MM (Torre Guaceto, Italy).

Figure S3. EICs of identified compounds of *A. vera* extracts with their MS/MS fragmentations

Table S1. Relative area of the compounds identified through LC-MS analysis. Data are shown as an average of three replicates and the relative standard deviation.

Class	Name	CA (Area)		CAN (Area)		E (Area)		MM (Area)		PO (Area)	
		Average	St. Dev	Average	St. Dev	Average	St. Dev	Average	St. Dev	Average	St. Dev
Alkaloids	Conessine	4388293	809453	532511	26548	5568470	954952	5947538	2870243	3433070	1724018
Alkaloids	Coniine	1388082	450451	98117792	15667237	433808	315767	1085342	364873	14375008	6590974
Alkaloids	(-)-Lupinine	198570	6691	3905843	1371256	235125	38786	191272	13636	1505693	375296
Alkaloids	Quinolizidine	4028850	331803	607225	161965	4498663	233370	5786497	2676615	6156770	654089
Alkaloids	Trigonelline	2811924	1532469	1992329	1216830	1889846	597831	4212120	972113	1082557	50587
Alkaloids	Tropine	304498	17052	1415936	817048	339110	62648	310523	169792	7945391	2778060
Alkaloids	Tropinone	283611	62305	2766603	230961	218879	8792	265503	66955	5154992	1561984
Amino acids	DL-Glutamine	422021680	41497305	188375061	98277724	51717528	36472196	282657532	243272941	8086012	1822618
Amino acids	Isoleucine	599601842	43200055	432421306	16995130	283548381	46187133	556804663	92708678	210386611	5449702
Amino acids	L-Methionine	2235279	264598	16586532	8651344	1764690	131782	3606251	613446	1589557	177140
Amino acids	L-Phenylalanine	694170284	13802480	361202120	46508483	374907994	161760480	700681487	245040987	157381928	4561121
Amino acids	Phenylalanylphenylalanine	4772359	2162730	4525383	1900338	1330237	291761	3433815	1066753	290677	84708
Amino acids	L-Proline	16680257	3717424	17837626	7216275	7295980	1621641	19042332	3078194	3968055	1554554
Amino acids	D-Pyroglutamic Acid	893479476	208639506	333458475	186508718	101774778	11405932	539196241	365973730	44674016	6474318
Amino acids	Pyroglutamyl-histidyl-glycine	1116887	258228	869646	138808	501004	33552	975074	302617	1014140	385764
Amino acids	N-Tetradecanoylaspartic acid	587866	64440	697286	263290	5102829	7600362	383107	87570	2543484	859036
Amino acids	D-Tryptophan	181773573	119946042	41875654	10300079	53563665	25300035	210239027	188214204	6737991	378799
Amino acids	L-Tyrosine	97767705	3994484	98452415	13508581	43938638	9364462	94402104	21233570	32795368	5697509
Amino acids	L-Valine	157490907	14832400	89425788	57843281	55539327	38334687	137361225	97240090	28070870	1312908
Antraquinones	Aloe-emodin	1467920	1136197	2093824	1422113	308758	21624	518073	238407	38678865	5399656
Antraquinones	Aloeresin A	5932494	3879013	10490936	7731992	6614806	6404570	5552682	3262316	10551874	3825301
Antraquinones	Aloeresin B	158297	44120	6202966	4219719	672432	485122	417617	261690	2278754	959182
Antraquinones	Aloesin	4680662	346790	49765175	32180240	5874652	2105784	8323367	4451236	86814985	41469257
Antraquinones	Methyl-Aloesinyl-Cinnamate	544004	158683	60731311	26272090	669521	135470	530905	158498	20489843	7077454
Aromatics	4-Acetamidobenzoic acid	12378149	2164647	949035	199845	13052163	3052869	21647914	5395417	1255388	238255
Aromatics	4-Phenylaminobenzoic acid	2144684	1673249	595395	302095	158477	6893	1058961	633837	158926	2054
Aromatics	Coumarin	6001821	1321806	56578610	22295984	5113428	868209	8657160	2746964	84808631	9446530
Aromatics	Hypoxanthine	7992564	3293945	6845043	4802636	6480718	5042760	6406224	3468667	1565339	1241602
Aromatics	Styrene	262009	9578	5885915	2351299	292201	46287	247130	39496	1985338	127958
Fatty acids	Erucic acid	8658500	5758223	302626792	163323277	1624344	449880	1294194	659131	6844059	1402724
Fatty acids	α-Linolenic acid	6441704	2149322	5320224	3062582	8695634	1171254	16385101	5012013	5077740	1406160
Fatty acids	Palmitic Acid	456969392	101743378	448397330	262687846	578155302	384594553	361442900	47518931	541026865	388810214

Fatty acids	Palmitoleic Acid	17841941	6387368	23345733	13597306	26418445	8950119	56327289	23698314	8056748	1647989
Glycosides	1′,2′-Dihydro-ϕ,ψ-caroten-1′-yl 6-O-tridecanoyl-β-D-glucopyranoside	25987301	23737441	1480473	49400	1408003	24021	1478169	72999	12666748	1301546
Glycosides	β,β-Caroten-3-yl 6-O-(11-methyldodecanoyl)-β-D-glucopyranoside	5209902	3513472	957571	64787	2051071	134426	1886204	303860	1412517	168804
Glycosides	Convallagenin A 3-O-α-L-arabinopyranoside	6232300	5233287	715284	11608	928258	397199	740557	81246	6721927	375544
Glycosides	Cucurbitacin S	12060409	16692932	278831	13353	268936	17184	451632	335313	328710	16788
Glycosides	Ethyl glucuronide	786190	501297	2308950	1369676	373130	21054	413597	59118	476894	33429
Glycosides	Ginsenoside Rg1	3565276	1272692	1042641	33338	7306517	1035978	6689050	2116490	3387975	874332
Glycosides	Saponarin	160500	3932	7526523	4014937	192643	31112	153159	8812	4195124	1032283
Organic acids	3-Dehydroquinic acid	5238270	3019123	17152045	6857213	3317609	1320374	6285128	2281367	961044	233085
Organic acids	Citraconic acid	2920585	182913	12343186	2098022	3791643	2063691	5160937	2266073	1075541	349139
Organic acids	Citramalic acid	5344023	66402	28232570	3828863	5185167	1798954	9916517	4579585	764064	137328
Organic acids	Citric acid	61068541	15293733	52028538	12100557	84863447	22618460	54652587	22664808	282886545	86043719
Organic acids	Eicosadienoic acid	14032466	13791680	635376	38845	1390273	1363156	598483	8172	11186532	3674761
Organic acids	Gabapentin	1796227	136512	8900563	3799353	2258362	90004	2126482	75436	3159287	248947
Organic acids	Gentisic acid	2265062	712826	2475312	221073	3274469	1284762	2358601	1401057	4242683	204466
Organic acids	Homogentisic acid	1639680	976425	1062847	89335	622158	89012	541536	50311	2198850	267762
Organic acids	Jasmonic acid ME	9995704	809430	73656388	36454365	10879571	1758456	17227625	9359225	46966303	758804
Organic acids	α-Ketoisovaleric acid	314664979	54709661	62557638	73164226	370925580	37149690	337479699	45578719	452972784	97055044
Organic acids	Malic acid	1729459712	627924774	2717503375	184170309	1304811462	408052742	963414228	222903805	641091512	87714541
Organic acids	Oxoglutaric acid	4373661	1468708	7145640	2136486	4746071	1045234	4338735	1888654	17382551	7781755
Organic acids	Pantothenic acid	286979293	94333752	560128864	131536099	138172735	24938886	514542667	86884344	55001368	15659433
Organic acids	Quinic acid	4775863	1867368	31696776	1602493	2560922	263456	3834544	1941123	22353926	7302745
Organic acids	Sinapic acid	177091	68996	1743005	302425	269552	65753	339828	218464	1661457	525566
Phenols	2,4,5-Trimethoxycinnamic acid	1481007	721785	712124	465340	1743753	1080993	1911140	289487	718728	302207
Phenols	3',4'-Dimethoxyflavone	332822	64192	3523110	2230204	1457712	924377	649266	273503	501619	232021
Phenols	4-Methoxycinnamic acid	1225857	124007	6617639	1212364	1235646	261400	1293782	380209	4830444	1859782
Phenols	Apigenin	4344345	2414710	456776	186768	1159737	1206647	3376173	1191302	8945137	2603806
Phenols	Chlorogenic acid	16602852	3498894	33947145	19509348	19488554	10605433	22715346	14582992	73320149	31243643
Phenols	Cinnamic acid	3747291	151428	2249362	313844	2355726	735961	3601277	1072242	1049204	180522
Phenols	Ethyl cinnamate	2130809	58309	3861613	1638430	3837053	447323	3017884	1148572	2881950	1283262
Phenols	Eupatorin	19913438	5721160	6008364	3943270	36046910	18865815	31011584	8671920	4908784	1191641
Phenols	Isopentyl cinnamate	11280722	6939863	713859	288516	38628059	9154334	30915331	4679394	34909711	14132361
Phenols	Tyramine	1917848	1043701	11102798	4669194	2401519	515478	2282196	1605938	14506852	756086
Steroids	Cortisol	8444703	6345157	2009841	191379	11170983	1665623	17659613	12862938	7989161	3704506

Sugars	Erythrose 4-P	39744778	17458008	5218257	736766	44475785	11404300	42371362	6845394	18902457	4540365
Sugars	Fructosyl valine	231594521	60797495	31161574	34952688	83838489	21040876	258555173	139495564	11881250	542650
Sugars	Galactaric acid	2201202	1513327	418219	143433	240387	68752	223029	13532	3445048	296737
Sugars	Glucose 1-P	53391799	11452177	10099483	3562065	83765107	10899082	80219725	10705815	20370128	808189
Sugars	D-(+)-Glucose	22677683	5050367	70191899	87885343	15359061	7799508	11094459	3181921	13447642	4320236
Sugars	Sucrose oleate	10371695	4027118	7462325	284539	6081022	538145	5845000	375402	14144140	87652
Terpenes	β-Damascenone	192048	75836	6242104	2389557	254989	27699	302881	70692	1530897	214639
Terpenes	(R)-Limonene	22142254	16028365	4639069	858354	13118077	5760088	87387976	25180110	7412293	4331831
Vitamins	Ascorbic acid	46756732	16212144	13794469	3117830	49592007	48154728	82257773	79839105	2876412	1036302
Vitamins	Biotin	1780564	762371	7888801	632018	971921	457658	3255617	380525	204812	33544
Vitamins	α-Tocopherol	6524696	5090309	1220838	476416	942094	35351	945649	38124	3882594	1281391

Figure S1. Description of the workflow used in Compound Discoverer 3.3, including key nodes and parameters used to filter compounds.

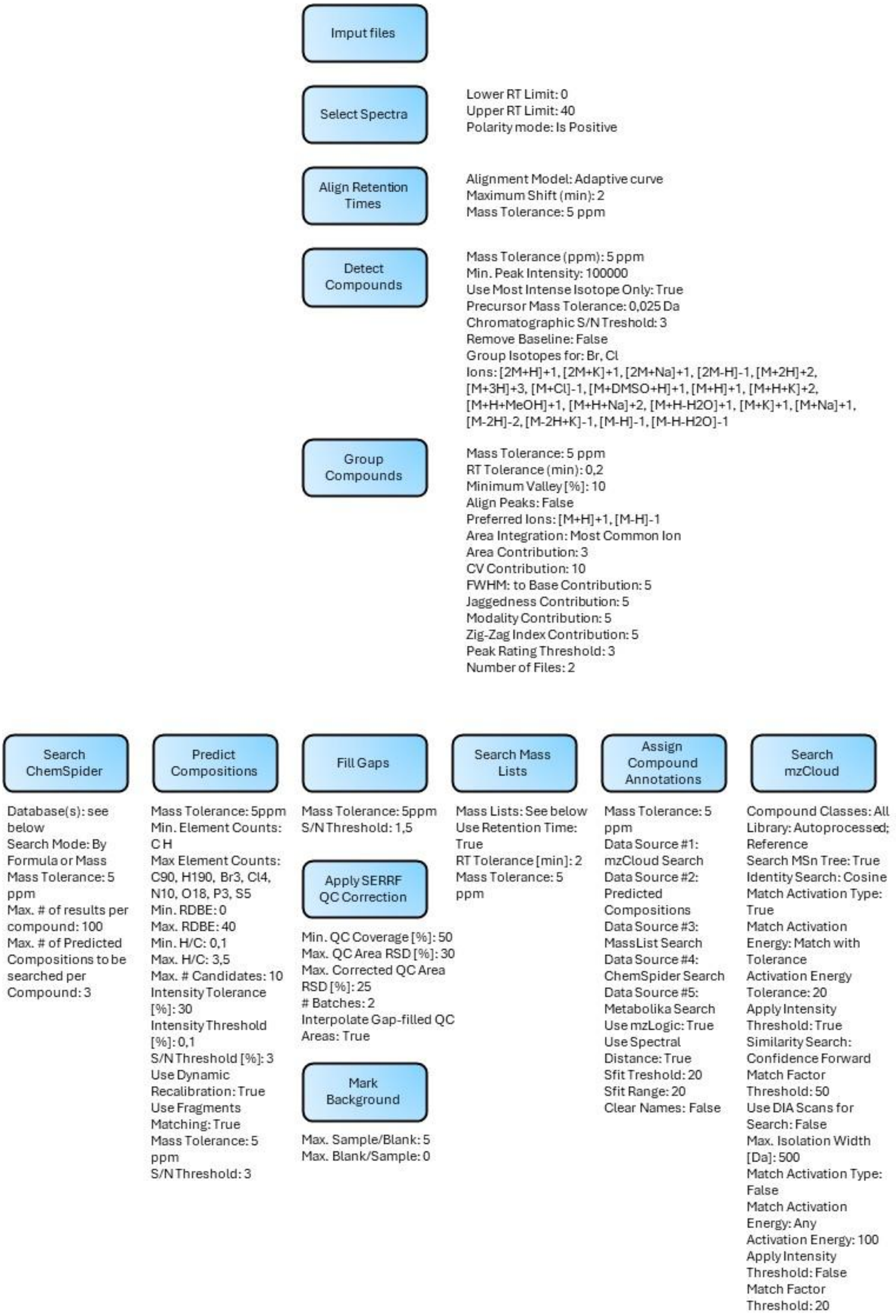

Figure S2. Representative LCs of *A. vera* extracts: PO (Portici, Italy); CA (Torre Guaceto, Italy); CAN (Gran Canaria, Spain); E (Torre Guaceto, Italy); MM (Torre Guaceto, Italy).

PO

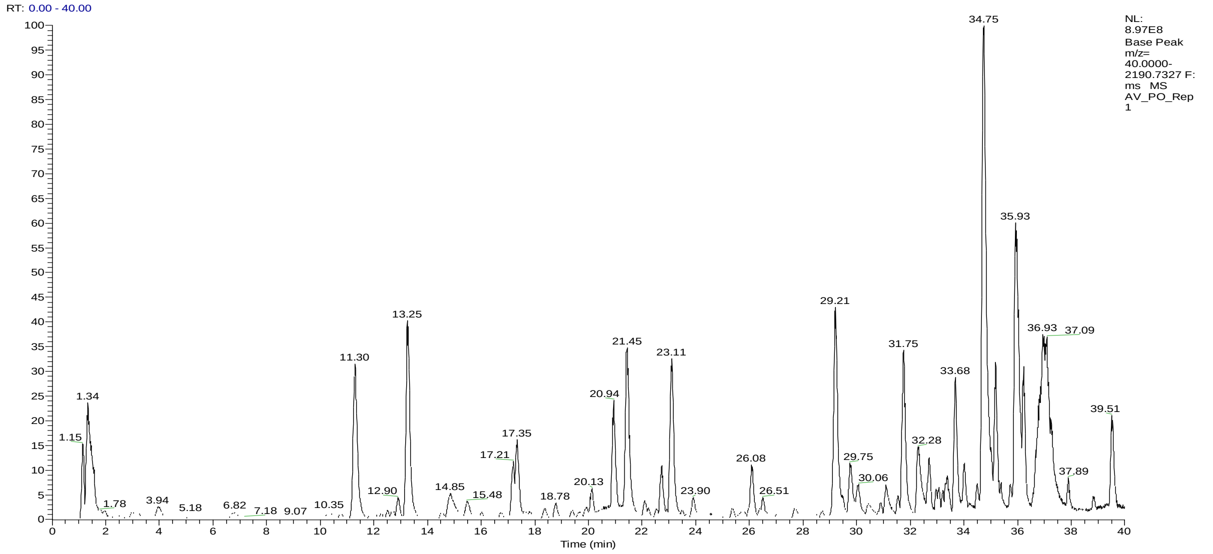

CA

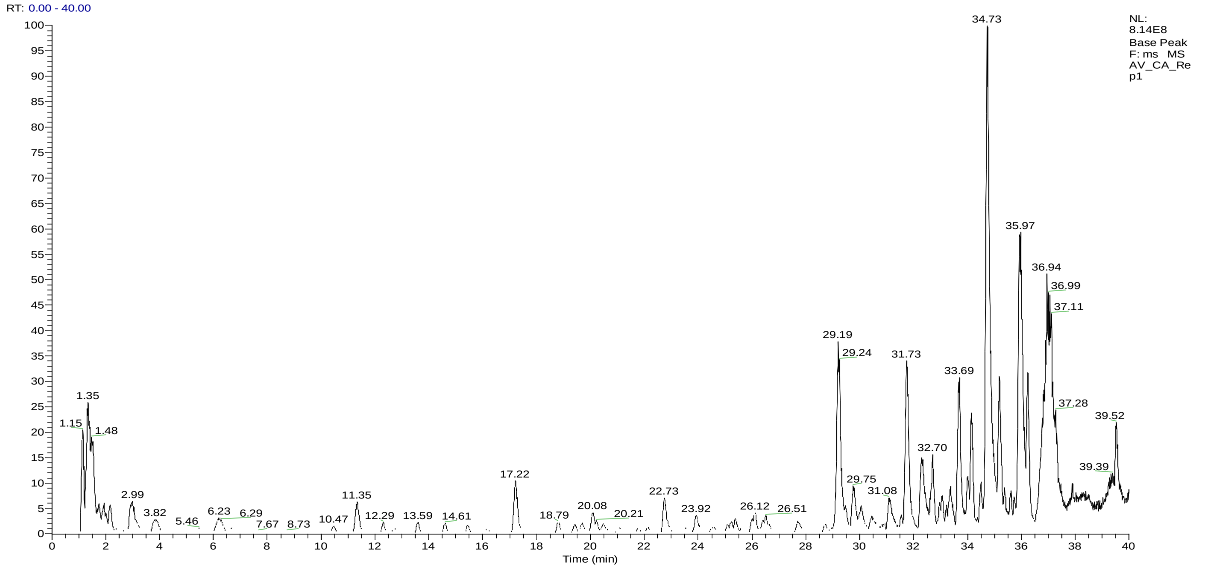

CAN

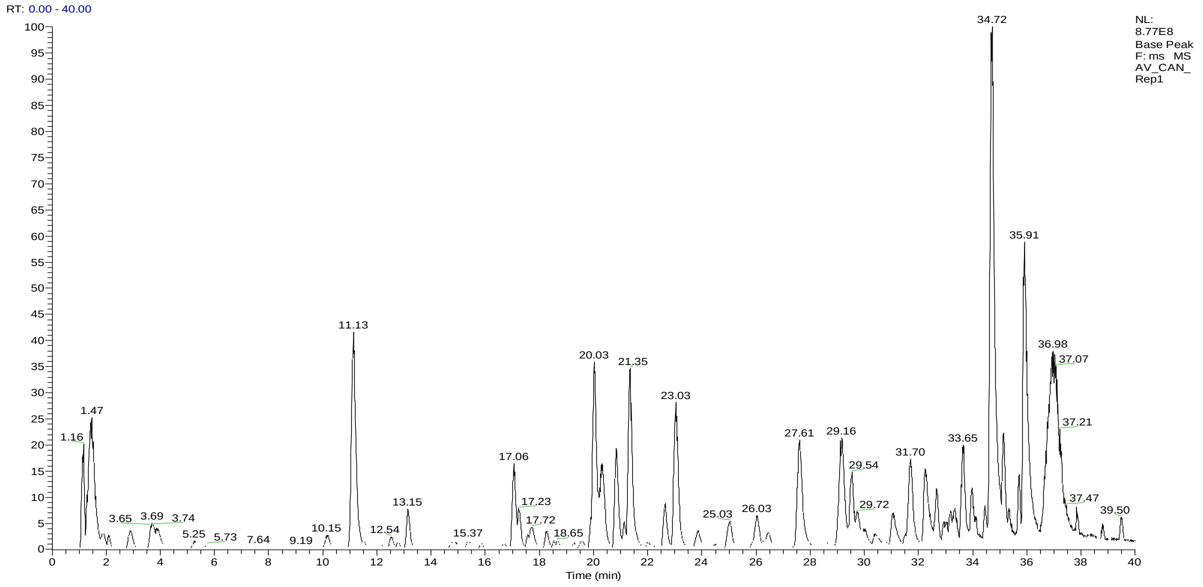
RT: 0.00 - 40.00
NL:
8.77E8
Base Peak
F: ms MS
AV_CAN_
Rep1
34.72
35.91
36.98
37.07
37.21
37.47
39.50
33.65
31.70
29.16
29.54
29.72
27.61
26.03
25.03
23.03
21.35
20.03
17.06
17.23
17.72
18.65
15.37
13.15
12.54
11.13
10.15
9.19
7.64
5.73
5.25
3.65
3.69
3.74
1.16
1.47
Time (min)

E

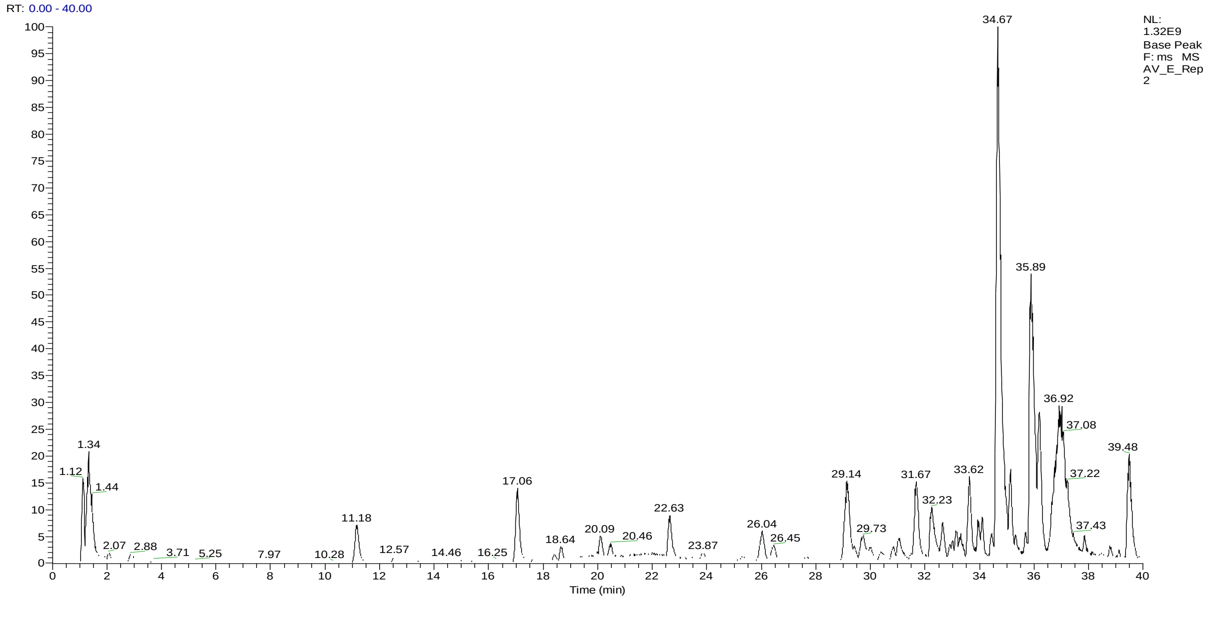
RT: 0.00 - 40.00
NL:
1.32E9
Base Peak
F: ms MS
AV_E_Rep
2
34.67
35.89
36.92
37.08
37.22
37.43
39.48
33.62
31.67
32.23
29.14
29.73
26.04
26.45
23.87
22.63
20.09
20.46
18.64
17.06
16.25
14.46
12.57
11.18
10.28
7.97
5.25
3.71
2.88
2.07
1.12
1.34
1.44
Time (min)

MM

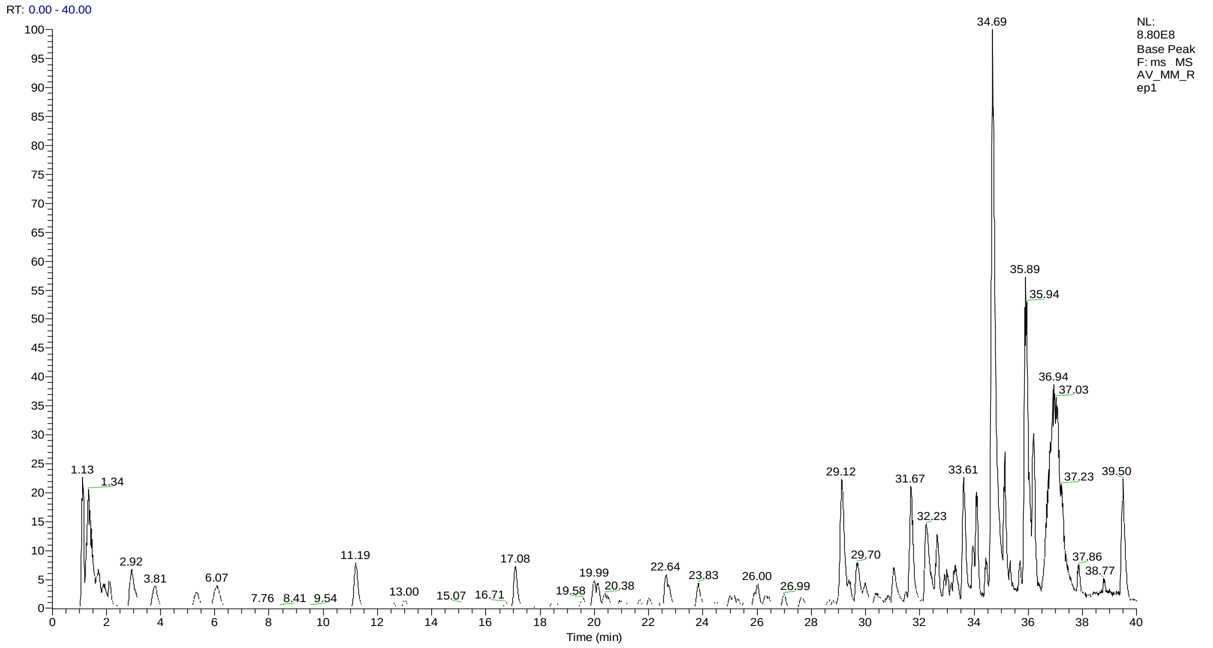
RT: 0.00 - 40.00
NL:
8.80E8
Base Peak
F: ms MS
AV_MM_R
ep1
Time (min)
34.69
35.89
35.94
36.94
37.03
37.23
39.50
29.12
31.67
33.61
32.23
29.70
37.86
38.77
1.13
1.34
2.92
3.81
6.07
7.76
8.41
9.54
11.19
13.00
15.07
16.71
17.08
19.58
19.99
20.38
22.64
23.83
26.00
26.99

Figure S3. EICs of identified compounds of *A. vera* extracts with their MS/MS fragmentations

DL Glutamine

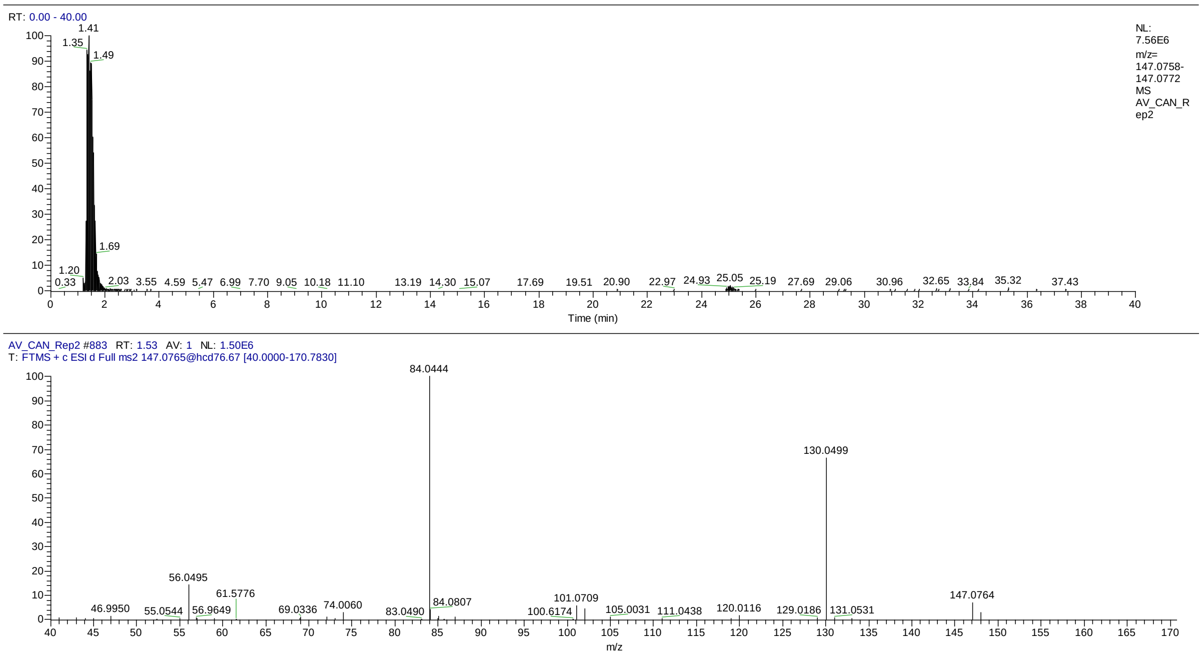

Isoleucine

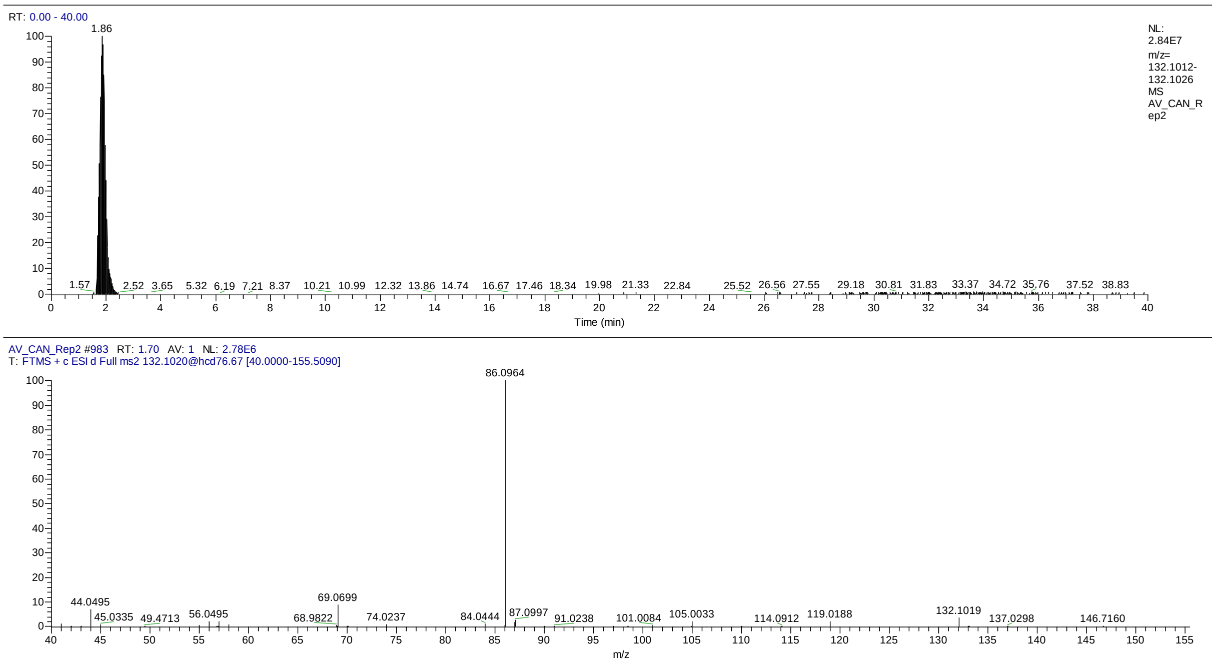

L-Phenylalanine

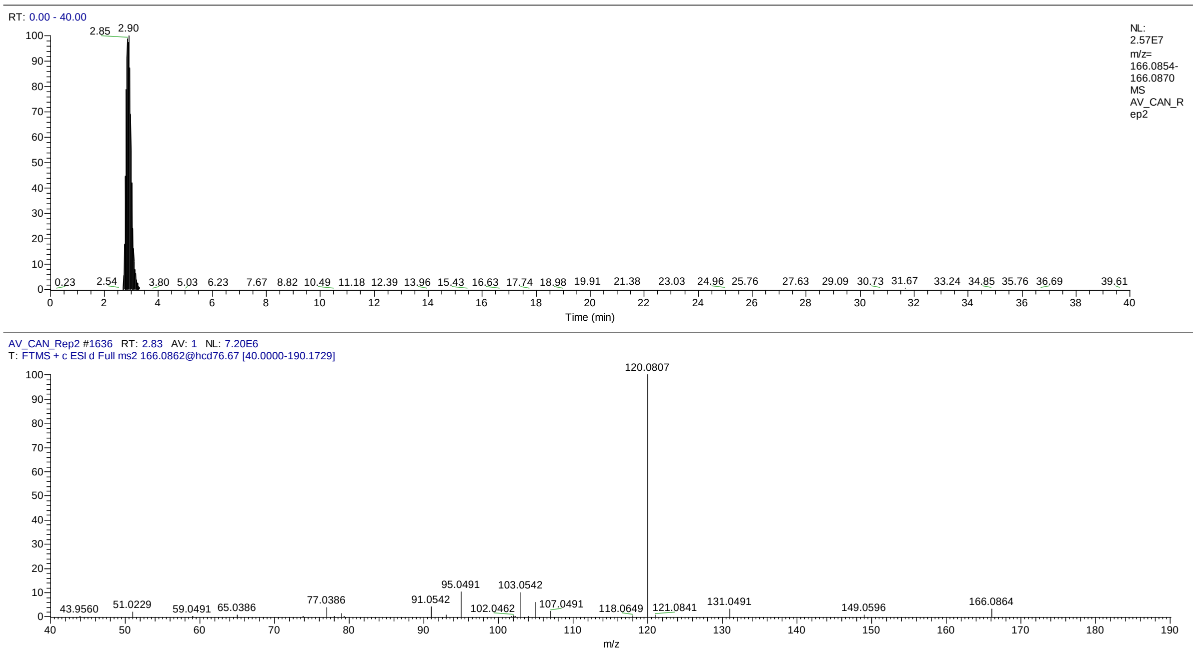

L-Proline

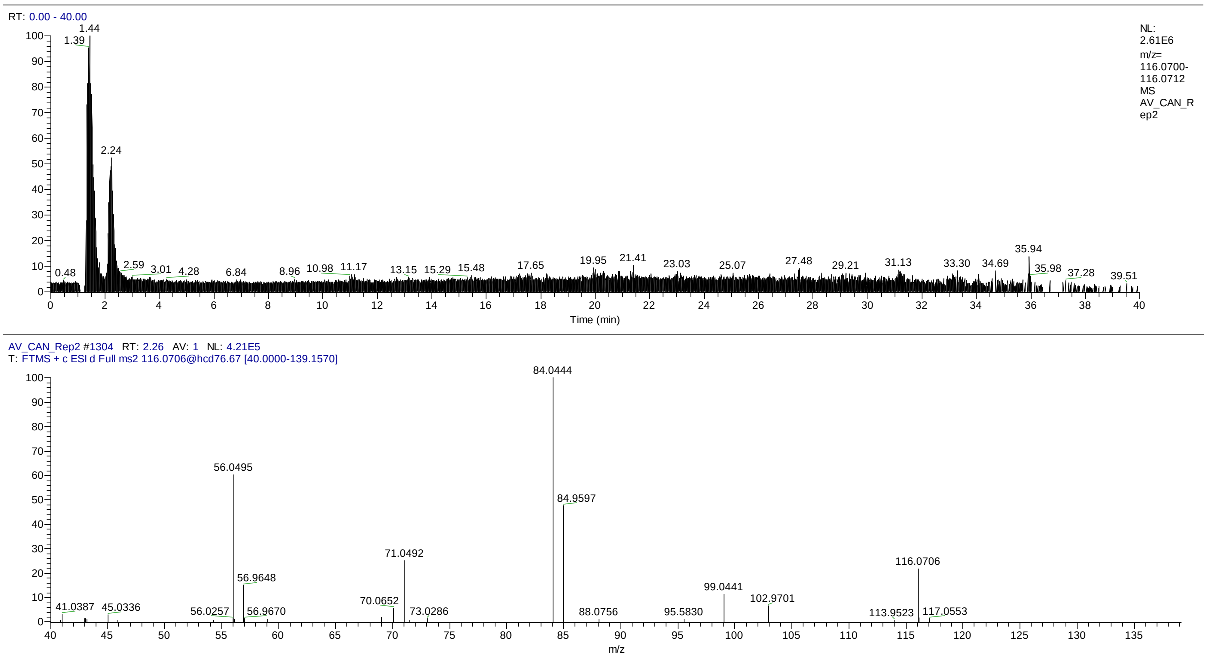

D-(+)-Pyroglutamic Acid

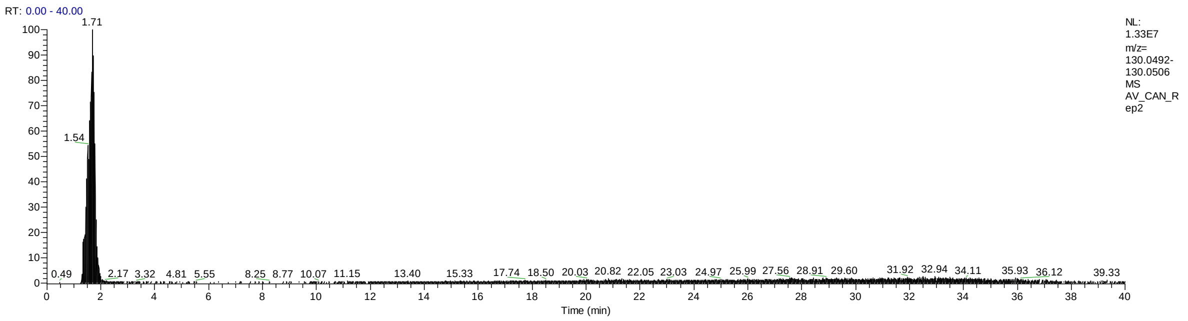

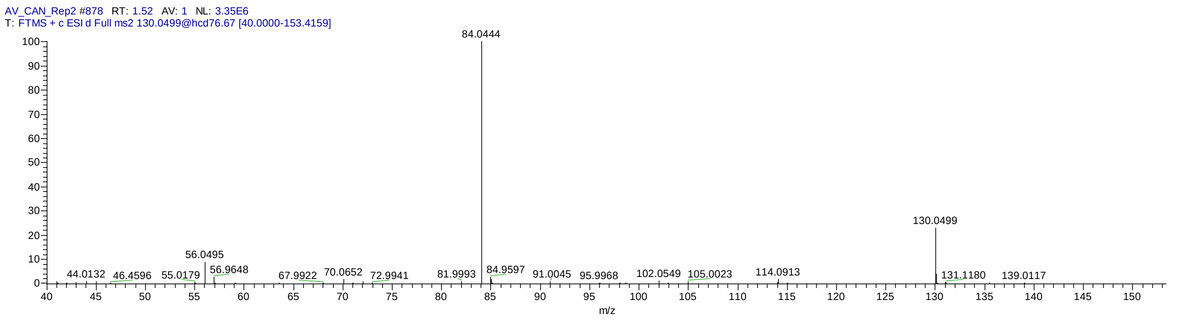

D-(+)-Tryptophan

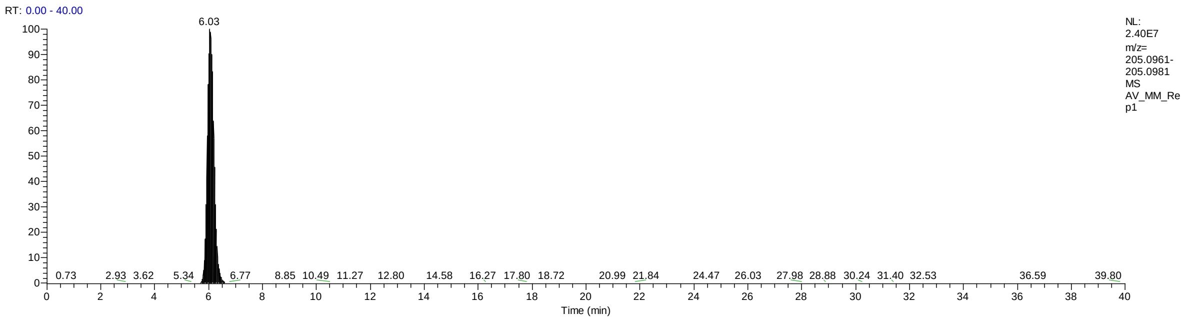

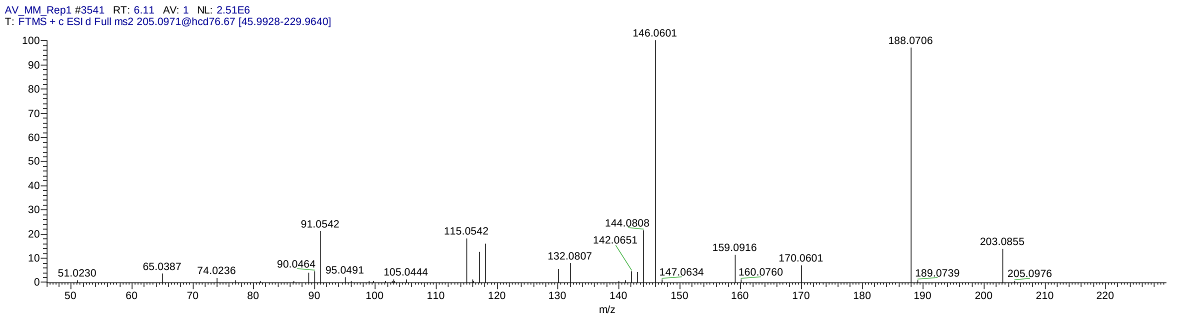

L-Tyrosine

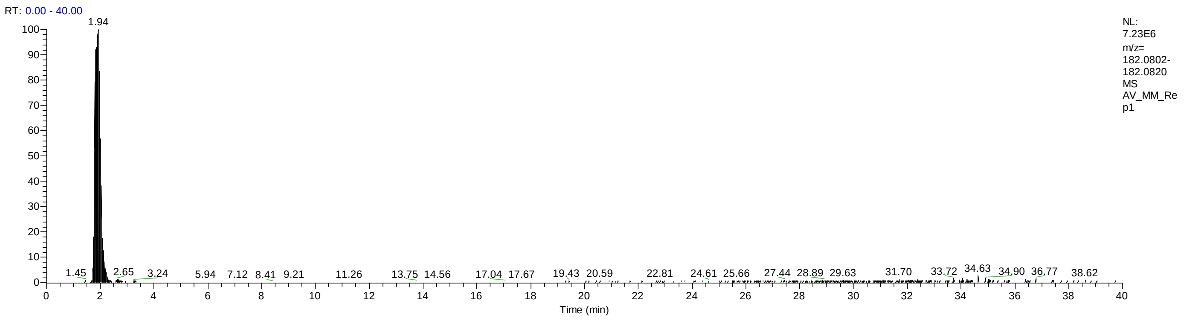

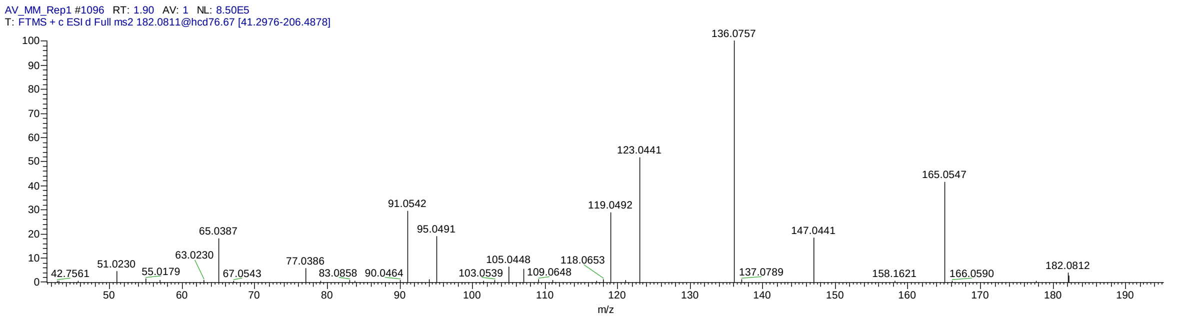

L-Valine

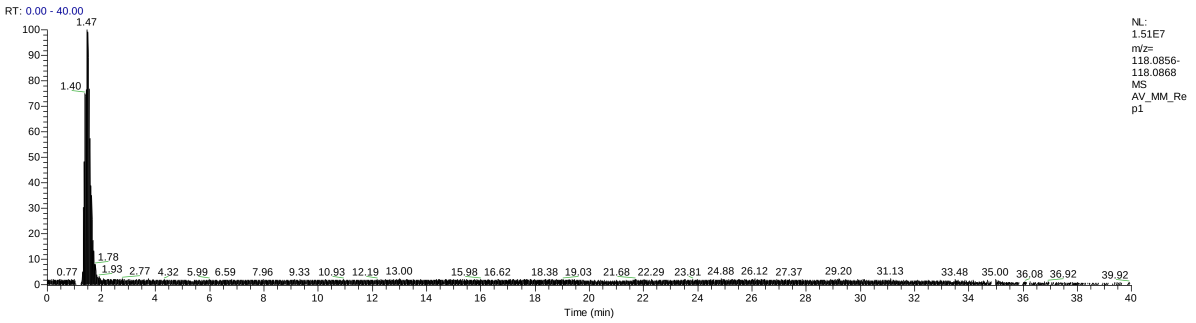

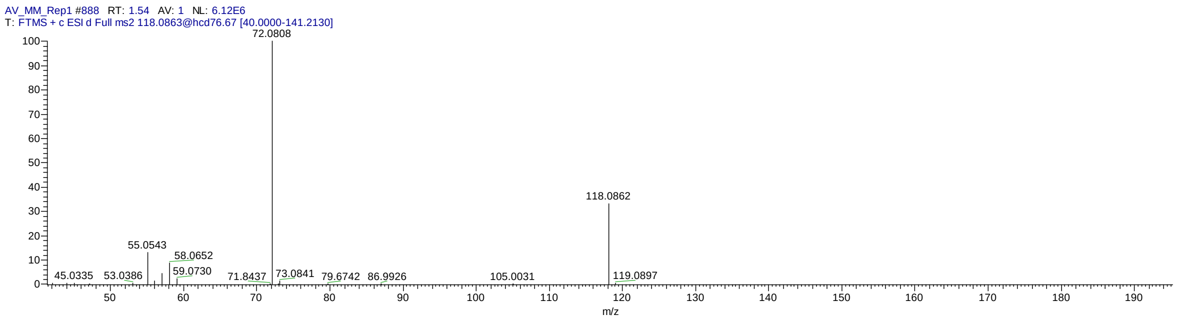

Aloe-emodin

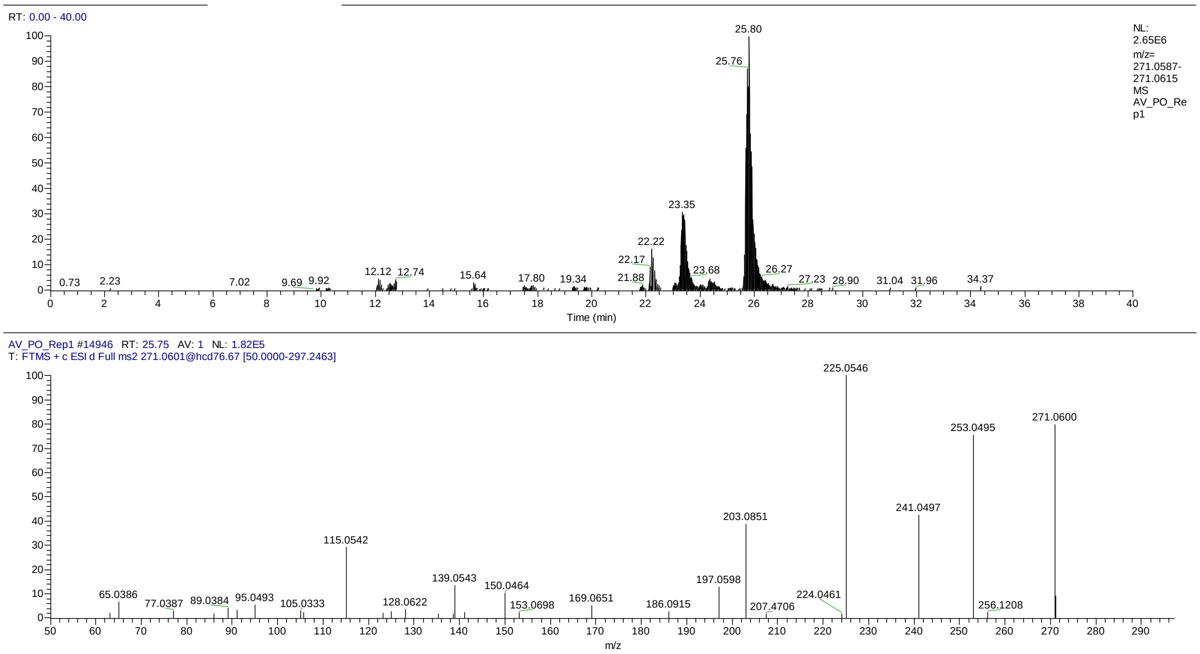

Aloeresin A

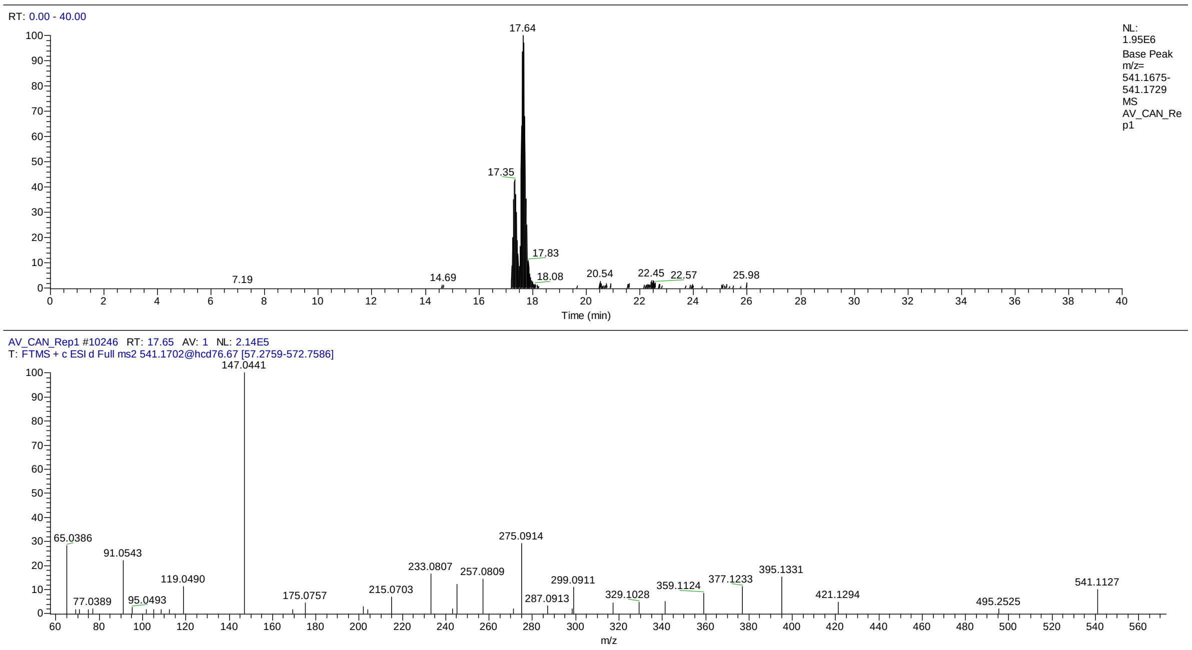

Aloeresin B

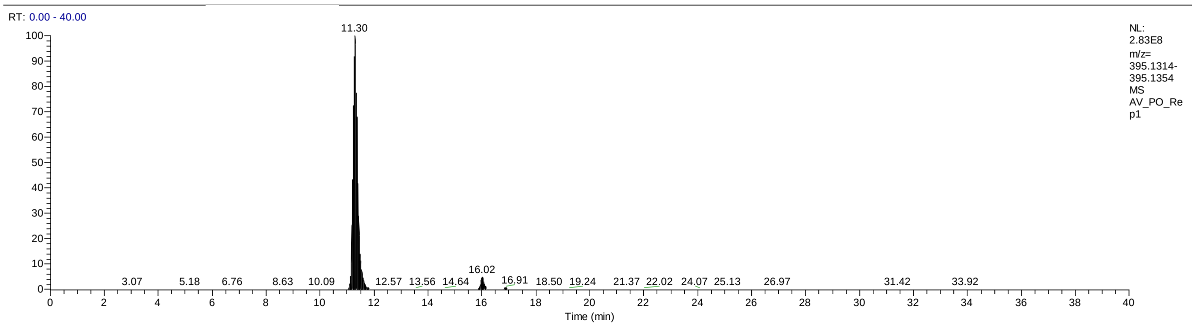

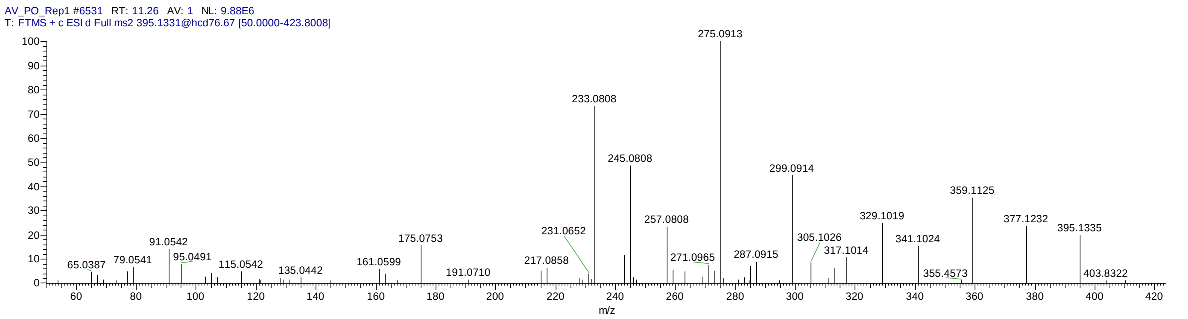

Aloesin

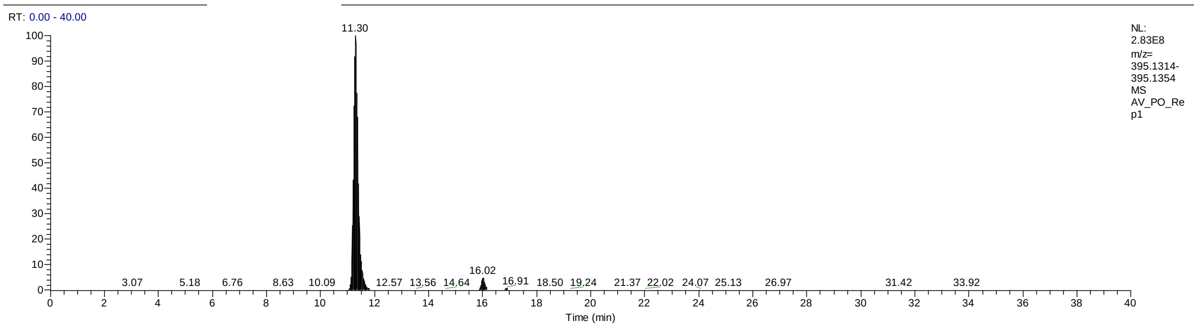

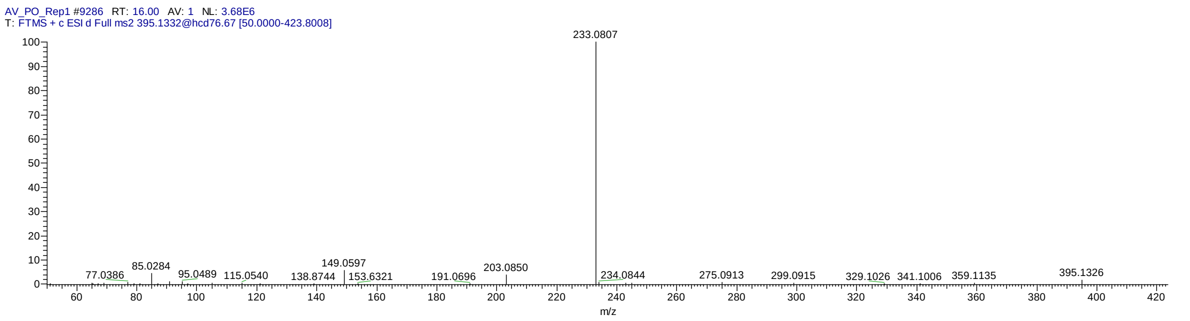

Methyl Aloesinyl Cinnamate

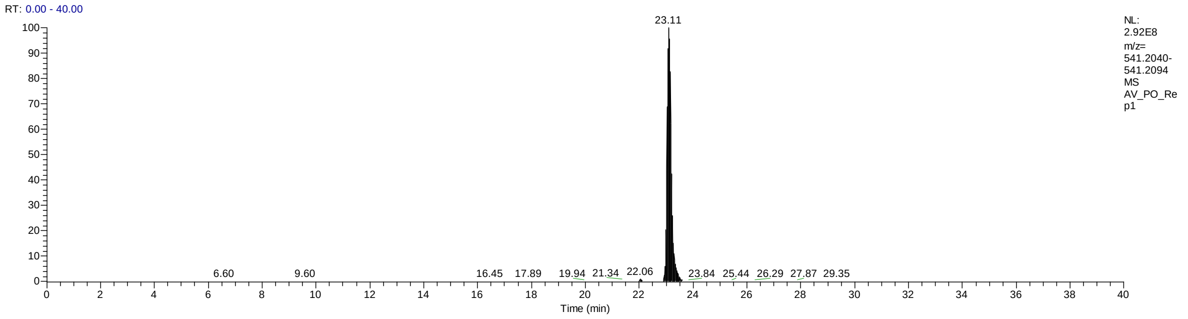

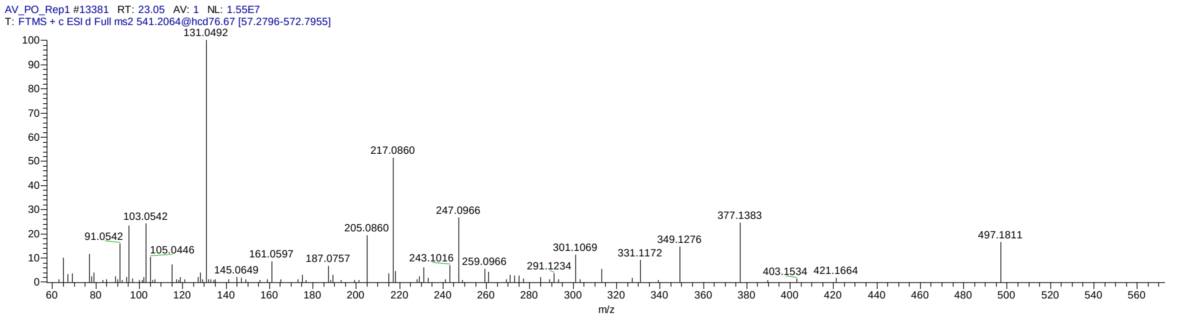

Coumarin

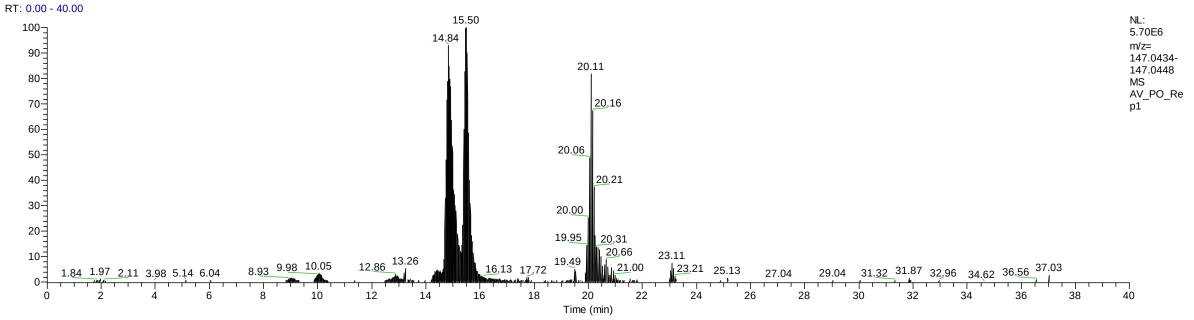

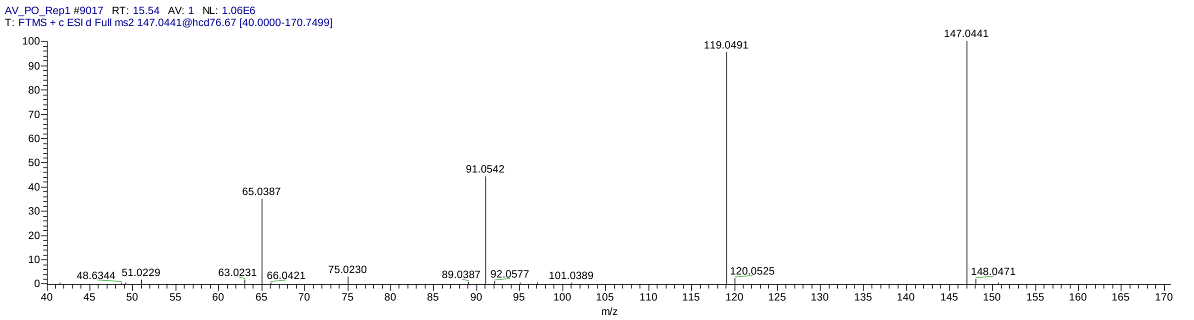

Hypoxanthine

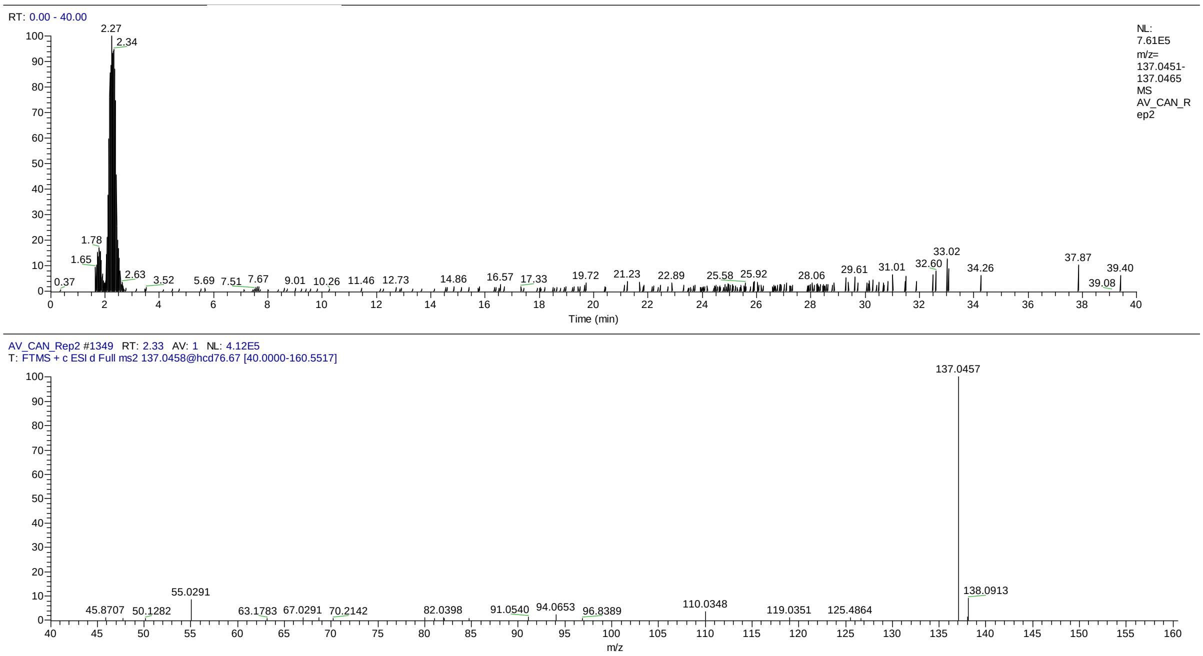

Styrene

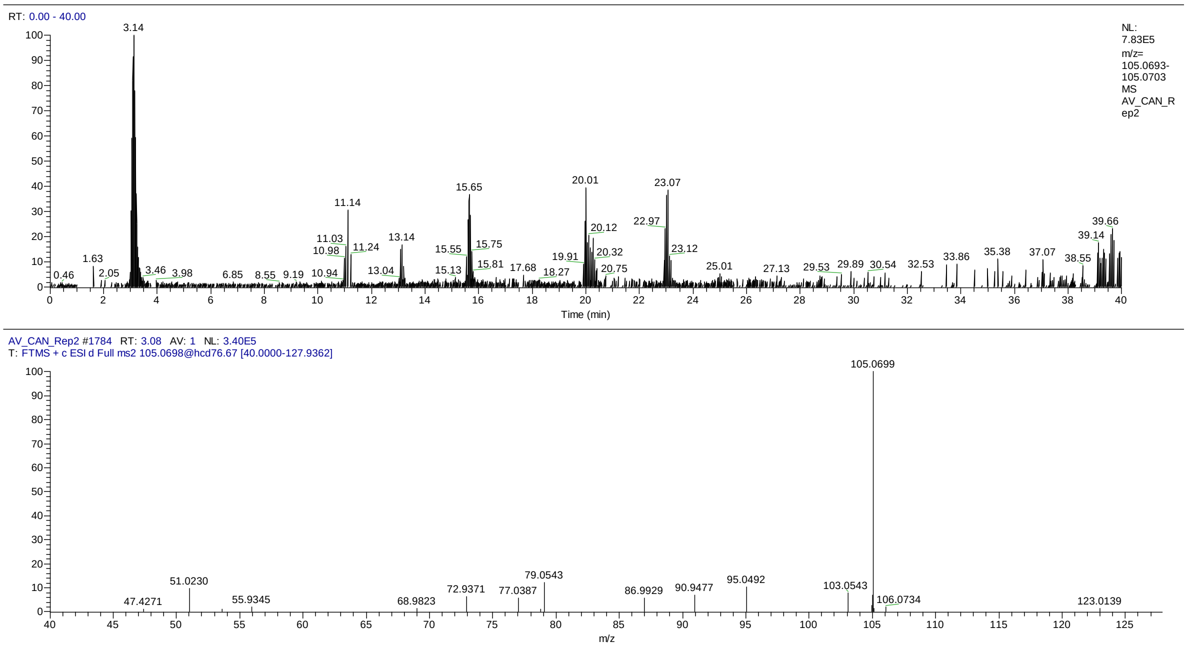

Erucic acid

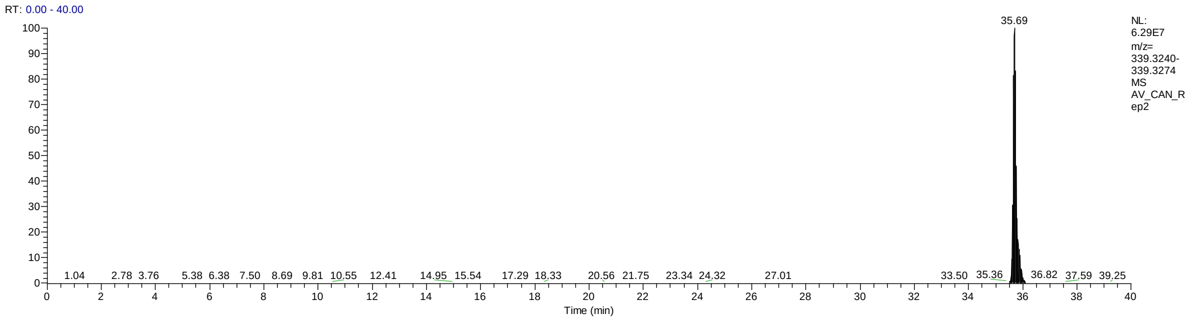

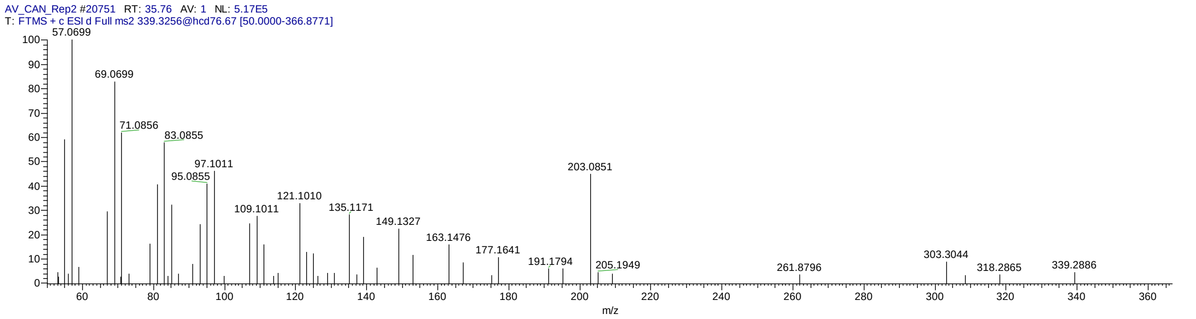

Palmitic acid

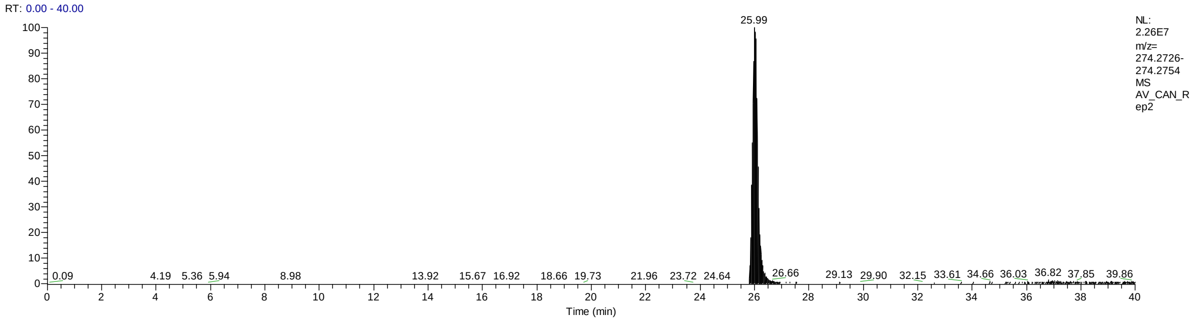

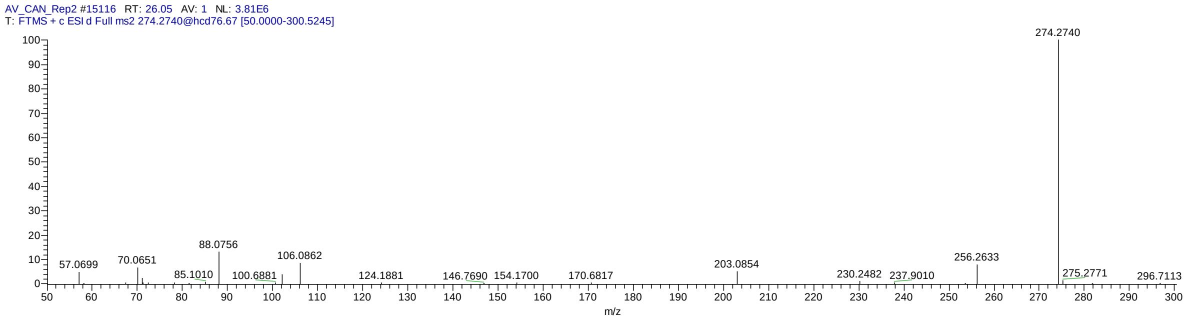

Palmitoleic acid

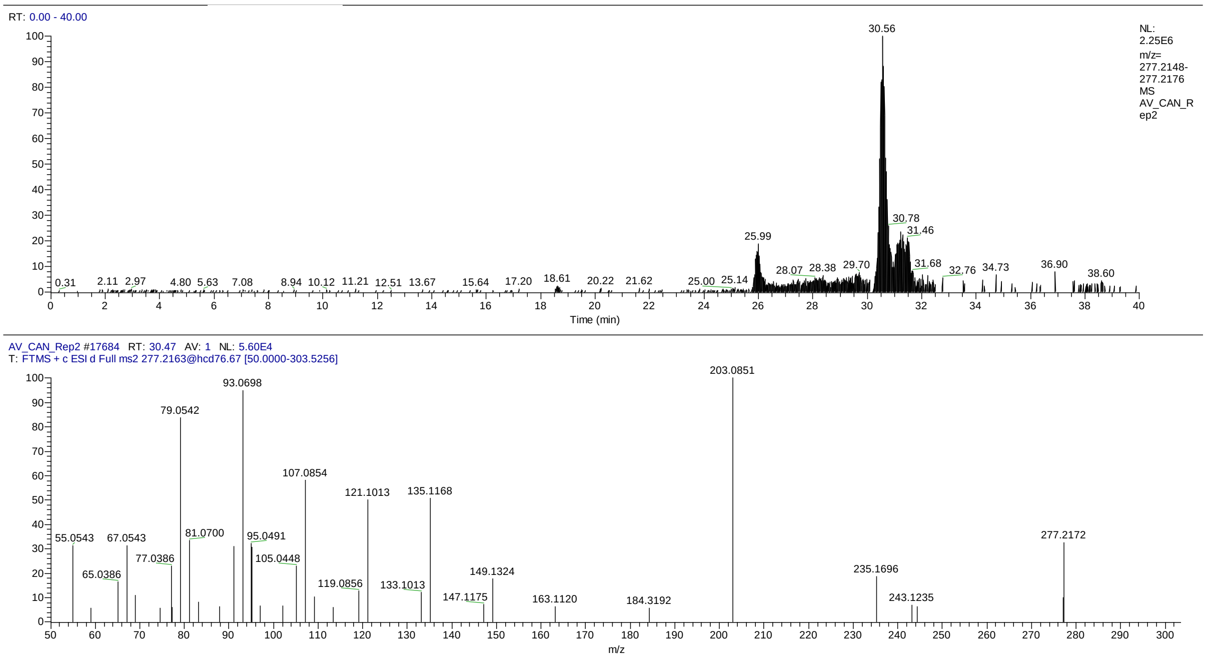

Cucurbitacin S

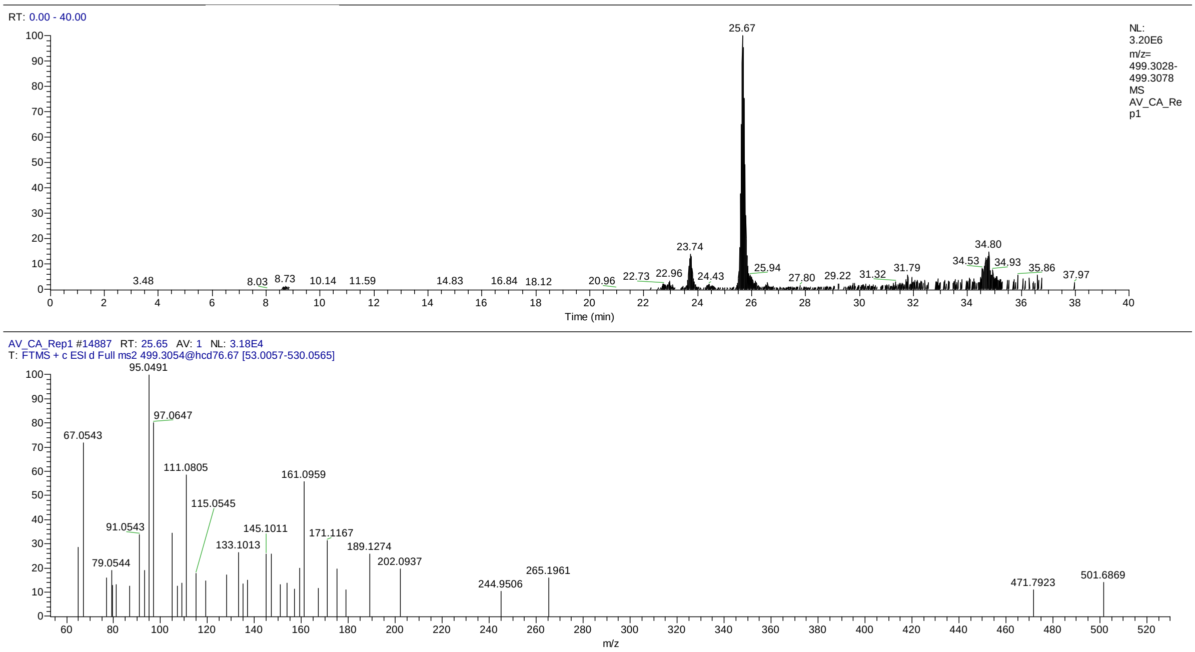

Citramalic acid

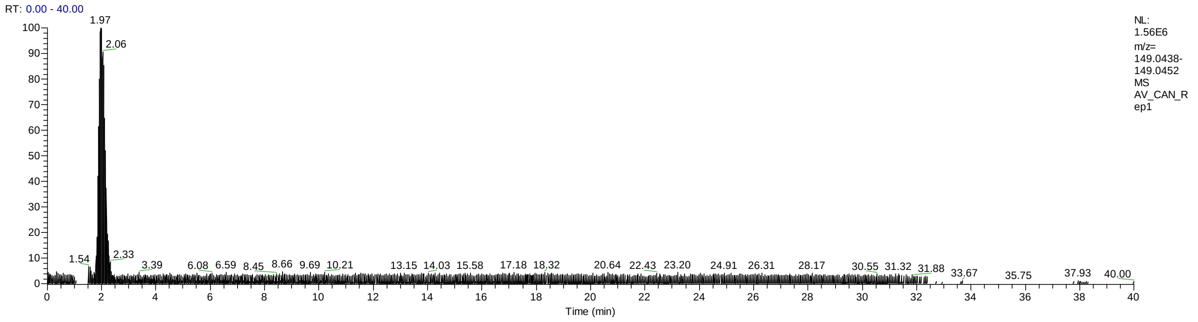

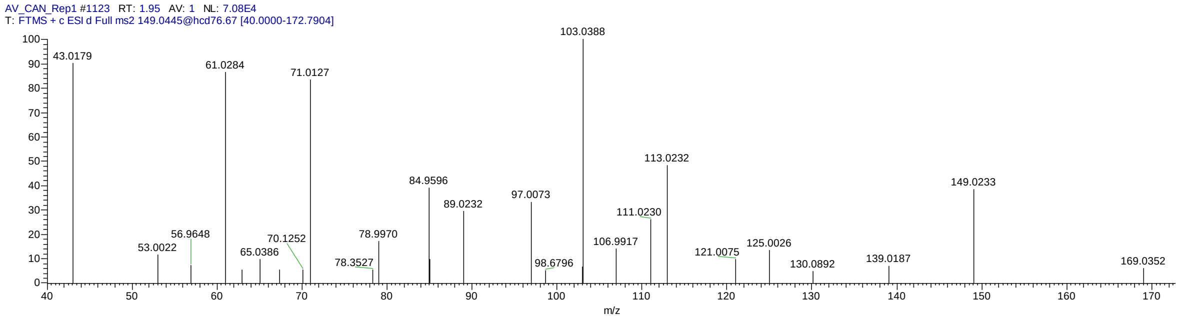

Citric Acid

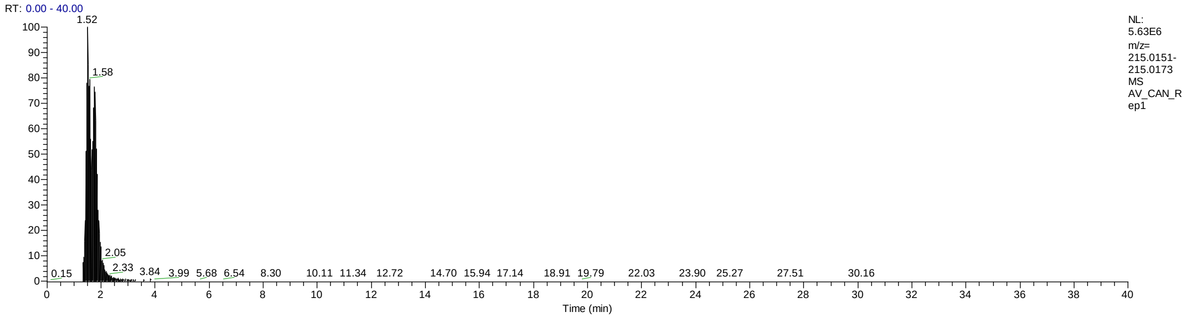

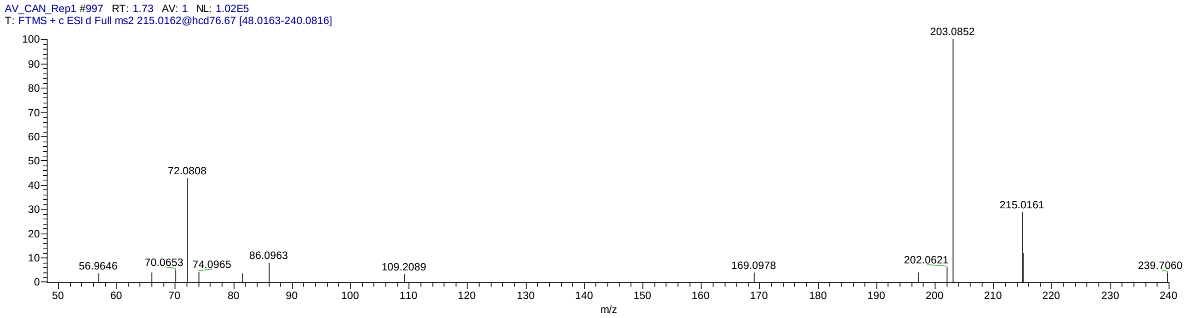

Jasmonic acid methyl ester

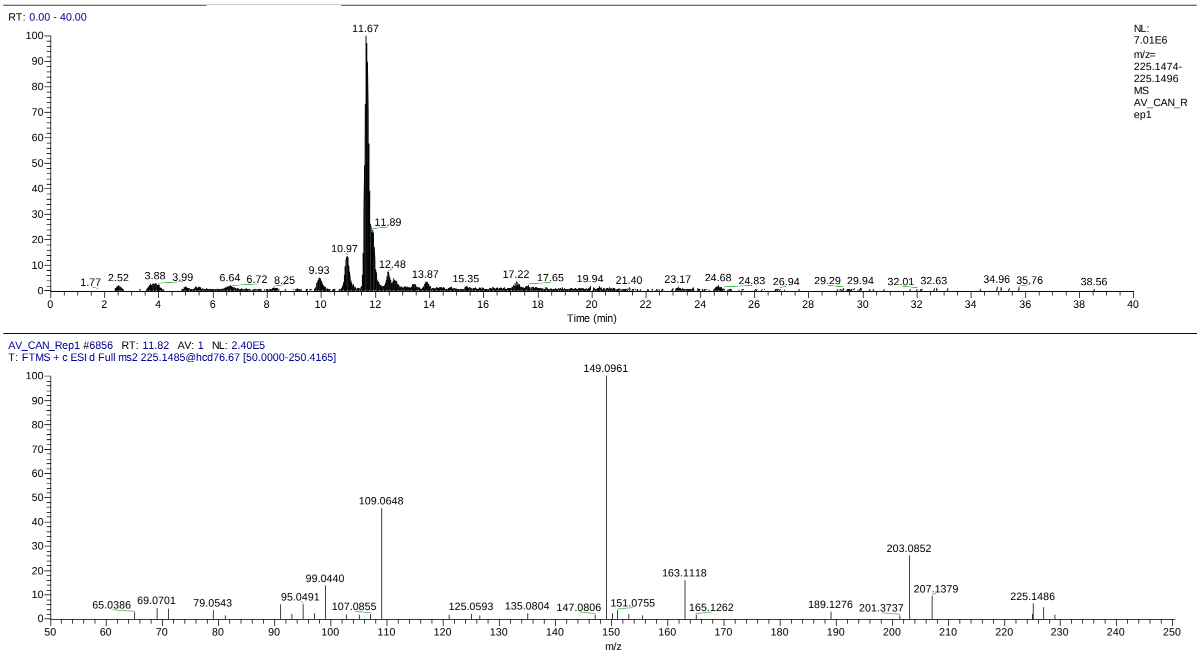

Pantothenic acid

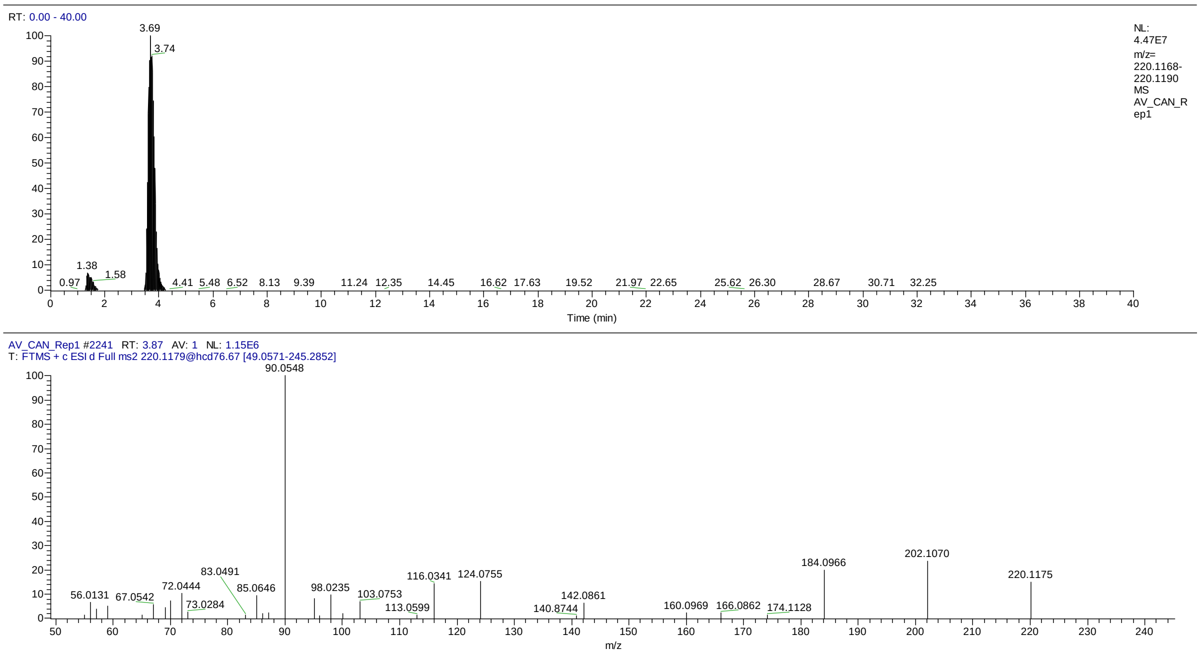

Chlorogenic acid

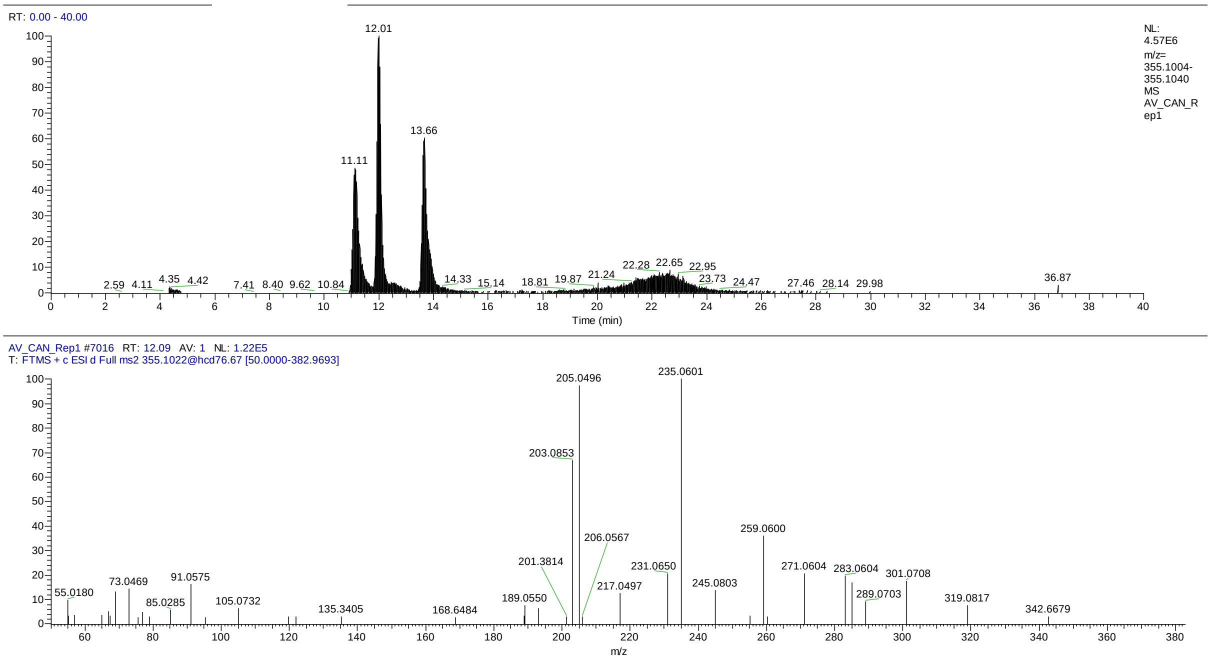

3’,4’-Dimethoxyflavone

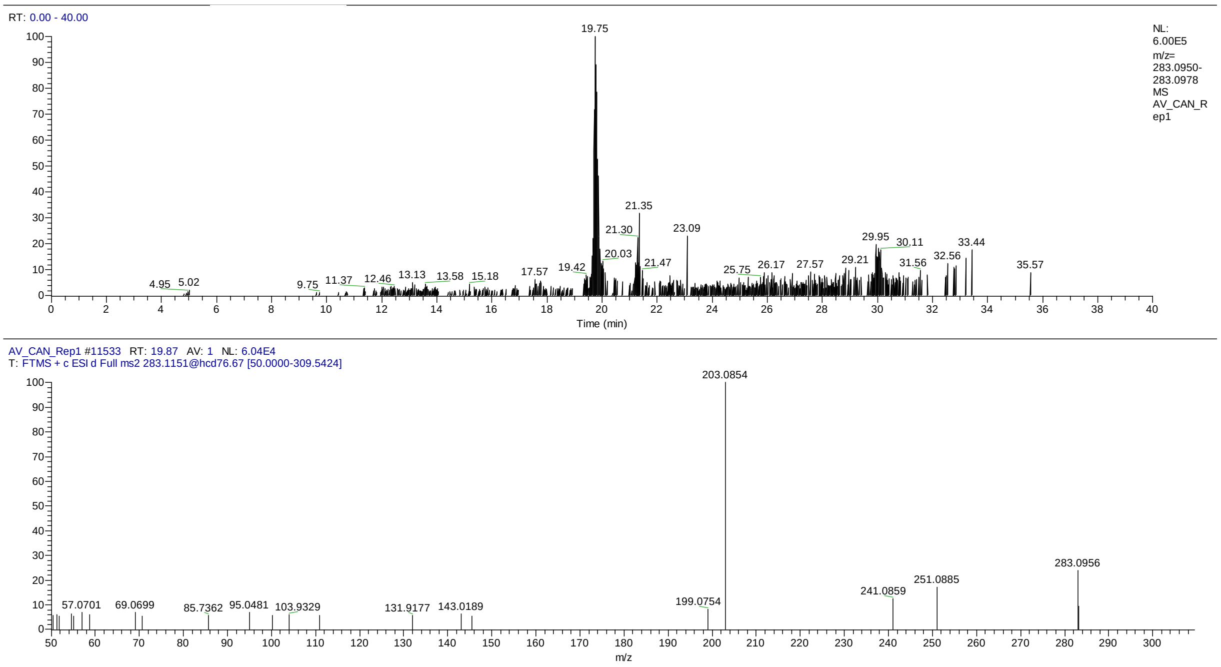

Supplement: Supplementary file 1 [file plants-14-01685-s001.zip › plants-3590617-supplementary.pdf]
